# Heterogeneity in Dental Tissue–Derived MSCs Revealed by Single-Cell RNA-seq

**DOI:** 10.1177/00220345241271997

**Published:** 2024-09-26

**Authors:** C. Behm, O. Miłek, K. Schwarz, A. Kovar, S. Derdak, X. Rausch-Fan, A. Moritz, O. Andrukhov

**Affiliations:** 1 Competence Center for Periodontal Research, University Clinic of Dentistry, Medical University of Vienna, Austria; 2 Core Facilities, Medical University of Vienna, Vienna, Austria; 3 Clinical Division of Conservative Dentistry and Periodontology, University Clinic of Dentistry, Medical University of Vienna, Austria; 4 Center for Clinical Research, University Clinic of Dentistry, Medical University of Vienna, Austria

**Keywords:** mesenchymal stromal cells, periodontal ligament, gingiva, single-cell analysis, transcriptomics, gene expression analysis

## Abstract

Mesenchymal stromal cells (MSCs) are multipotent, progenitor cells that reside in tissues across the human body, including the periodontal ligament (PDL) and gingiva. They are a promising therapeutic tool for various degenerative and inflammatory diseases. However, different heterogeneity levels caused by tissue-to-tissue and donor-to-donor variability, and even intercellular differences within a given MSCs population, restrict their therapeutic potential. There are considerable efforts to decipher these heterogeneity levels using different “omics” approaches, including single-cell transcriptomics. Previous studies applied this approach to compare MSCs isolated from various tissues of different individuals, but distinguishing between donor-to-donor and tissue-to-tissue variability is still challenging. In this study, MSCs were isolated from the PDL and gingiva of 5 periodontally healthy individuals and cultured in vitro. A total of 3,844 transcriptomes were generated using single-cell mRNA sequencing. Clustering across the 2 different tissues per donor identified PDL- and gingiva-specific and tissue-spanning MSCs subpopulations with unique upregulated gene sets. Gene/pathway enrichment and protein-protein interaction (PPI) network analysis revealed differences restricted to several cellular processes between tissue-specific subpopulations, indicating a limited tissue-of-origin variability in MSCs. Gene expression, pathway enrichment, and PPI network analysis across all donors’ PDL- or gingiva-specific subpopulations showed significant but limited donor-to-donor differences. In conclusion, this study demonstrates tissue- and donor-specific variabilities in the transcriptome level of PDL- and gingiva-derived MSCs, which seem restricted to specific cellular processes. Identifying tissue-specific and tissue-spanning subpopulations highlights the intercellular differences in dental tissue–derived MSCs. It could be reasonable to control MSCs at a single-cell level to ensure their properties before transplantation.

## Introduction

Mesenchymal stromal cells (MSCs) are important in maintaining tissue homeostasis, controlling inflammation, and tissue regeneration (Viswanathan et al. 2019). They are perivascularly located (Shi and Gronthos 2003; Sharpe and Yianni 2019) in various tissues, including the periodontal ligament (PDL) (Seo et al. 2004) and gingiva (Mitrano et al. 2010). PDL- and gingiva-derived MSCs comply with the minimal criteria for MSCs, including non-hematopoietic surface markers expression, a trilineage differentiation potential, and immunomodulatory abilities (Andrukhov et al. 2019; Viswanathan et al. 2019). Clinical trials already test the transplantation of ex vivo propagated MSCs, but the clinical application of MSC transplantation is still limited due to various factors (Levy et al. 2020; Costa et al. 2021), including different heterogeneity levels.

MSCs from different sources exhibit variability on various “omics” levels, such as the transcriptome (Levy et al. 2020; Costa et al. 2021). This variability manifests in tissue-to-tissue and donor-to-donor (Costa et al. 2021) differences regarding their proliferation (Li et al. 2015), differentiation (Heo et al. 2016), and immunomodulation (Li et al. 2015). This heterogeneity can be attributed to varying donor, tissue source, and culture properties (Levy et al. 2020; Costa et al. 2021). Even individual cells within a population can exhibit variability in their omics profiles (Wang et al. 2021) with clonal differences in their basic properties (Russell et al. 2010).

Reducing this heterogeneity to increase the predictability of MSCs as a therapeutic tool is crucial. Therefore, it is essential to understand this heterogeneity using omics techniques on a single-cell resolution (Zhang, Xu, and Xu 2022). Previous studies have shown tissue-of-origin and intercellular heterogeneity on the transcriptome level using single-cell approaches (Barrett et al. 2019; Huang et al. 2019; Zhou, Lin, et al. 2019; Medrano-Trochez et al. 2021; Wang et al. 2021; Lee et al. 2022; Zhang, Han, et al. 2022; Xu et al. 2023; Yang et al. 2024). However, these studies compared MSCs isolated from different tissues without considering donor-to-donor variability.

The objective of this study was to explore tissue-to-tissue and donor-to-donor variabilities on the transcriptomic level between in vitro cultured MSCs isolated from the PDL and gingiva. The aim was to identify tissue-specific MSC subpopulations in the PDL and gingiva and compare their expression profiles. Doing this analysis on a donor-to-donor basis allowed us to exclude donor-to-donor variability in the intertissue analysis. The second aim was to compare the gene expression profiles of MSCs from different donors per tissue.

## Materials and Methods

An extended Material and Methods section (Supplemental appendix), information about the TotalSeq A (0251-0260) anti-human Hashtag antibodies (Appendix Table 1), and sequencing output (Appendix Tables 3 to 6) are provided as supplementary information.

Primary human MSCs were isolated from the PDL and gingiva, obtained from 5 extracted third molars. The teeth were extracted from 5 individuals (2 males and 3 females, aged between 17 and 22 y [Appendix Table 2]), obtaining 5 PDL- and gingiva-derived MSC samples from the same donors (*n* = 10). All patients, or their parents if applicable, signed an informed consent form. The Medical University of Vienna Ethics Committee approved the MSCs’ isolation and all experimental techniques (protocol No. 1079/2019). The study was conducted in compliance with the Declaration of Helsinki.

Cell cycle–synchronized MSCs were labeled with oligonucleotide-tagged antibodies. The cell cycle synchronization allowed exclusion of the cell cycle–dependent gene expression as a confounding factor and to focus on the donor’s genetic composition (Costa et al. 2021). Single-cell preparation was conducted following the 10x Genomics Single Cell Protocols – Cell Preparation Guide. scRNA-seq libraries were constructed by the Chromium Controller using the Next GEM Single Cell 3′ GEM, Library & Gel Bead Kit (v3.1, 10x Genomics, Pleasanton, CA, USA) according to the manufacturer’s instructions for a maximum cell recovery of 10,000 cells. Single-cell library sequencing was conducted using the Illumina NovaSeq 6000 platform. Demultiplexing and preprocessing of raw sequencing data were executed using the cellranger multi and count pipeline (v7.0.1., 10x Genomics). Hashtag oligonucleotides assigned the sample identity (tissue type and donor) to each cell barcode. The sequenced reads were aligned to the GRCh38-2020-A assembly version. The Loupe Browser (v6.3.0., 10X Genomics) was used to illustrate demultiplexed data among all 10 samples by t-distributed stochastic neighbor embedding (t-SNE) projections (Wang et al. 2021). Differential gene expression (DGE) analysis was performed using the Seurat (V4) package (Satija et al. 2015; Butler et al. 2018; Stuart et al. 2019; Hao et al. 2021) in RStudio (v4.2.3.). Cluster-specific cell barcodes and cluster annotations were extracted from the loupe file to filter the cellranger count pipeline output data in a cluster-specific manner, excluding clusters consisting of mitotic cells. To investigate functional differences between PDL- and gingiva-specific or between tissue-specific subpopulations of different donors, pathway and process enrichment analysis was conducted on highly differently (upregulated) expressed genes (p_val_adj < 0.05) using Metascape (Zhou, Zhou, et al. 2019). Metascape (Zhou, Zhou, et al. 2019) was used to individually apply protein-protein interaction (PPI) enrichment analysis to highly differently (upregulated) expressed genes for each subpopulation.

## Results

### Single-Cell Transcriptome Analysis of PDL- and Gingiva-Derived MSCs

**Figure 1.**
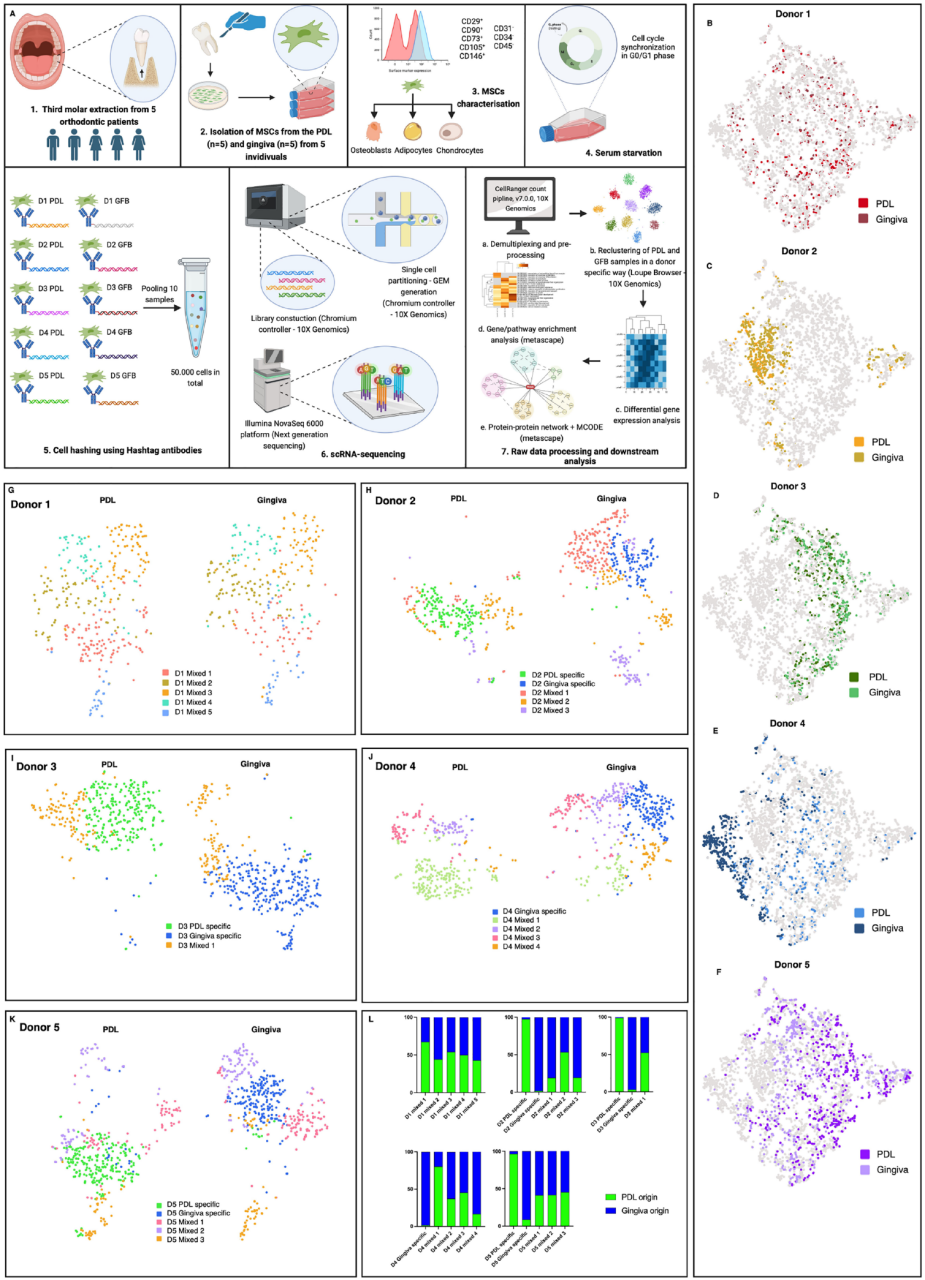
Periodontal ligament (PDL)– and gingiva-specific subpopulations in mesenchymal stromal cells (MSCs) derived from the PDL and gingiva. (**A**) Study workflow and experimental design (created with Biorender.com). (**B–F**) t-distributed stochastic neighbor embedding (t-SNE) projections with 3,844 single-cell transcriptomes of the PDL- and gingiva-derived MSCs labeled in different colors per donor. (**G–K**) t-SNE projections show each donor’s PDL- and gingiva-specific and mixed subpopulation. (**L**) The percentage composition of each subpopulation per donor (D1–D5) is based on the origin of the MSCs’ tissue. 90% was used as the cutoff value for annotating the subpopulations as PDL or gingiva specific.

PDL- (*n* = 5) and gingiva-derived (*n* = 5) MSCs were isolated from 5 individuals (donors 1 to 5), cultured in vitro, and processed for scRNA-seq analysis (Fig. 1A). The character of the MSCs of the isolated cells was confirmed by common criteria (Appendix Fig. 1 to 4).

Demultiplexing separated the hash-tagged MSCs (Appendix Fig. 5A), identifying 4,766 single-MSC transcriptomes. After excluding the barcodes without any cell multiplexing oligo and the multiplets (Appendix Fig. 5C), 3,844 MSCs were assigned to 1 of the 10 samples and included in further analyses (Appendix Fig. 5B, D). The median Unique Molecular Identifier (UMI) counts, genes, and reads per cell were similar throughout all PDL- and gingiva-derived MSC samples (Appendix Fig. 5E–G). The gender-specific expression of XIST (female-specific), RPS4Y1, and DDX3Y (male-specific) verified successful demultiplexing (Appendix Fig. 5H–J).

### Exploring Tissue-to-Tissue Heterogeneity

t-SNE projections with tissue-specific colored single-cell transcriptomes per donor (Fig. 1B–F) revealed a partial overlap of the PDL- and gingiva-specific transcriptomes within the donors, indicating restricted tissue-to-tissue variability. After removing low-quality MSCs by setting the threshold by UMI counts per barcode, gene counts per barcode, and mitochondrial UMIs for each clustering process per donor, PDL- and gingiva-derived MSCs were reclustered per donor to investigate the intertissue variability more sophisticatedly. The comparison of the expression of highly variable genes revealed PDL- and gingiva-specific subpopulations (>90% of MSCs from 1 tissue type) in donors 2, 3, and 5 (Fig. 1G–L), having similar numbers of MSCs in the tissue-specific subpopulations per donor (Appendix Fig. 5K–O). In donor 4, a tissue-specific subset was detected only in the gingiva, whereas in donor 1, no tissue-specific subpopulations were observed. MSC subpopulations that contained cells from both tissues (<90% of cells from 1 tissue type) were defined as mixed subsets (Fig. 1G–L). Comparable expression levels of MSCs’ surface markers were detected in the tissue-specific and mixed MSCs’ subsets (Appendix Figs. 6A–E and 7A–B). The log2 summed expression of osteogenic and adipogenic differentiation markers was comparable between all observed MSCs’ subsets per donor (Appendix Fig. 8A–B). The expression of chondrogenic differentiation markers was low.

**Figure 2.**
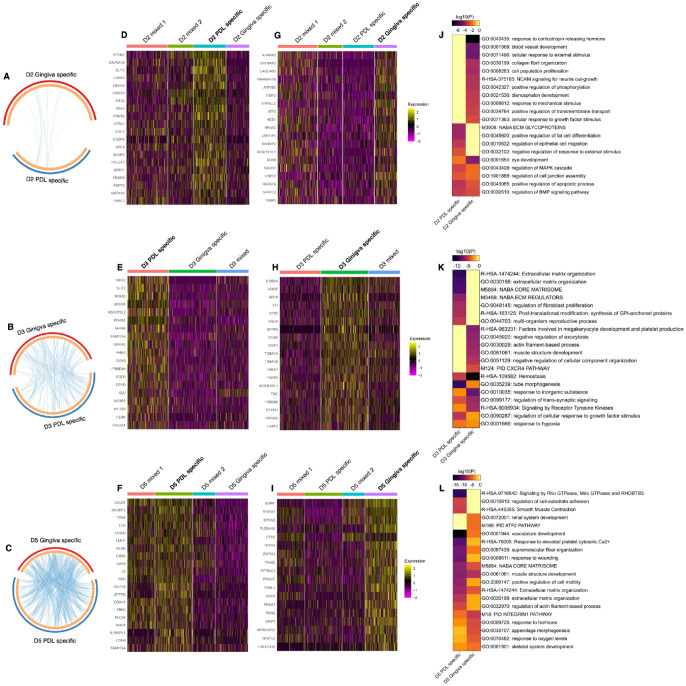
Differential gene expression (DGE) and gene/pathway enrichment analysis comparing periodontal ligament (PDL)– and gingiva-specific subpopulations. (**A–C**) Circos plot illustrates the functional overlap of significantly upregulated genes between the PDL- and gingiva-specific subpopulation in donors 2 (A), 3 (B), and 5 (C). The light orange arc per subpopulation depicts the uniqueness of the genes in each subpopulation. In contrast, the blue lines link genes between the subpopulations, which are different but share the same significantly enriched ontology terms. The lists of significantly upregulated genes in the PDL- and gingiva-specific subpopulations are presented by the blue and red arcs, respectively. (**D–I**) Gene expression heat maps depicting the top 20 differently expressed genes of individual cells between PDL- (D–F) and gingiva- (G–I) specific subpopulations in donors 2 (D, G), 3 (E, H) and 5 (F, I). Mixed subpopulations were excluded from the analysis. The yellow color demonstrates the upregulated while the pink color represents the downregulated gene expression values. (**J–L**) Enriched terms in the PDL- and gingiva-specific subpopulations are displayed by a heat map for donors 2 (J), 3 (K), and 5 (L). Each term represents a cluster of significantly enriched terms, identified by calculating the accumulative hypergeometric *P* values and enrichment factors and using the GO Biological Processes, Canonical Pathways, and Reactome Gene Sets databases. The enriched terms were hierarchically clustered by Kappa-statistical similarities between their included genes. The enriched term with the best *P* value was selected as a representative term for a given enriched cluster. The enrichment map is colored by the log10(*P*) values. The lack of an enriched term in one of the subpopulations is depicted in gray.

The pairwise DGE analysis revealed specific gene signatures for PDL- and gingiva-specific MSCs subpopulations in donor 2 (Fig. 2A), 3 (Fig. 2B), and 5 (Fig. 2C). This analysis was not performed for donors 1 and 4 because they did not exhibit tissue-specific subpopulations in both gingiva and PDL. PDL- and gingiva-specific MSC subpopulations have differently expressed gene signatures. The top 20 upregulated genes per tissue-specific subpopulations are depicted in heat maps per donor (Fig. 2D–I) and are described in the supplemental appendix (extended results version).

Pathway and process enrichment analysis identified numerous clusters, including terms enriched in PDL- and gingiva-specific MSC subpopulations. In addition, within the top 20 clusters, clusters of gene ontology terms were solely enriched in the PDL- or gingiva-derived MSC subpopulation (Fig. 2J–L and Appendix Fig. 9 to 11).

In donor 2, 4 and 11 clusters were solely enriched in the PDL- and gingiva-specific subpopulations, respectively (Fig. 2J and Appendix Fig. 9A–B). In donor 3, 7 and 6 solely enriched clusters were detected in the PDL- and gingiva-derived subpopulations, respectively (Fig. 2K and Appendix Fig. 10). In donor 5, 3 and 2 enriched clusters were explicitly identified for the PDL- and gingiva-derived subpopulations, respectively (Fig. 2L and Appendix Fig. 11).

**Figure 3.**
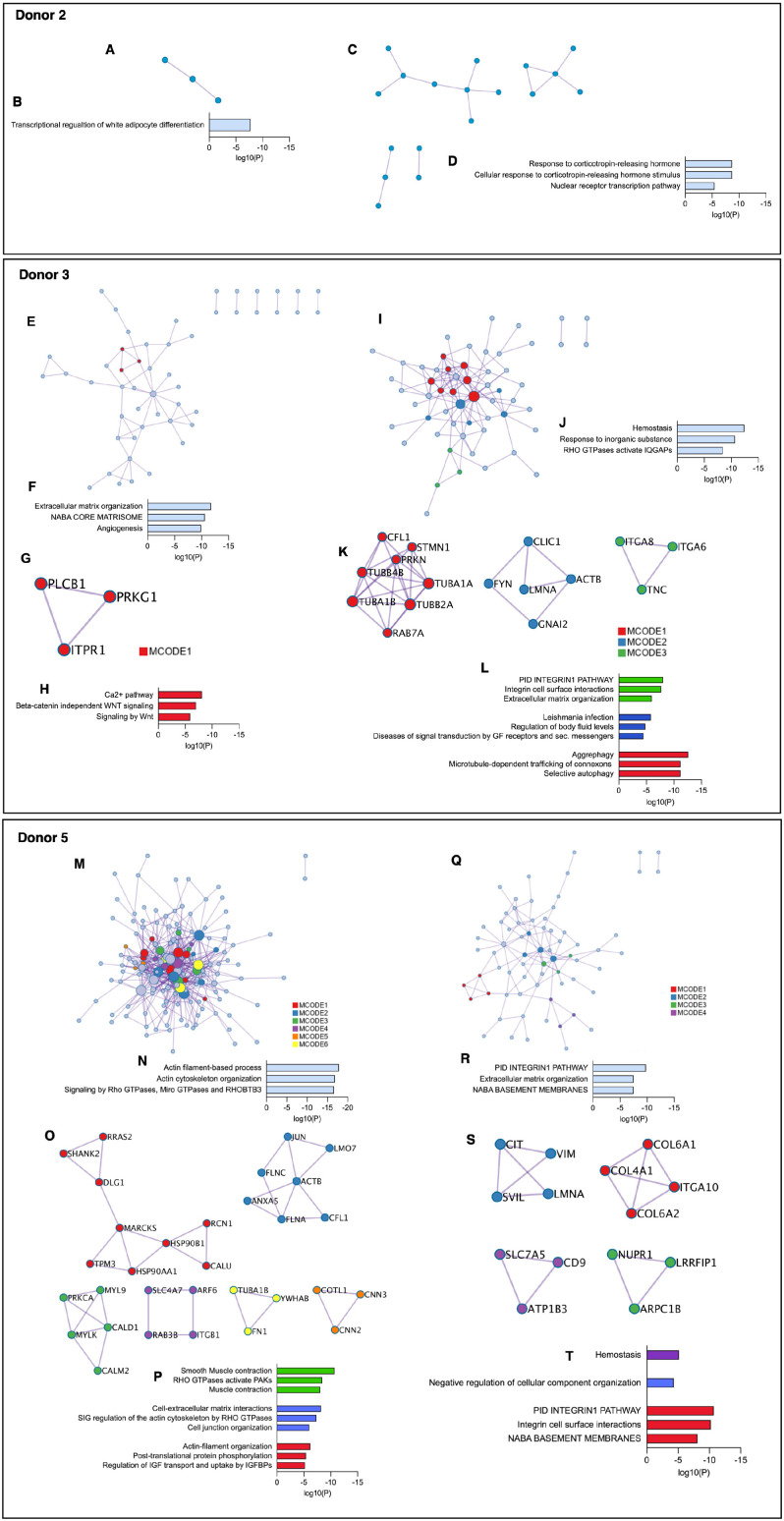
Protein-protein interaction (PPI) and Molecular Complex Detection (MCODE) identification within upregulated genes of tissue-specific subpopulations in donor 2 (**A–D**), 3 (**E–L**), and 5 (**M–T**). (A, C, E, I, M, O) Upregulated PPI networks identified within the periodontal ligament (PDL)– (A, E, M) and gingiva- (C, I, Q) specific mesenchymal stromal cell (MSC) subpopulations in donors 2, 3, and 5. Running the MCODE algorithm has not identified densely connected protein networks in donor 2. In donor 3, the MCODE algorithm identified 1 (MCODE 1) and 3 (MCODE 1–3) densely connected protein networks in the PDL- (G) and gingiva- (K) specific subpopulations, respectively. In donor 5, 6 (MCODE 1–6) and 4 (MCODE 1–4) densely connected protein networks were identified in the PDL- (O) and gingiva- (S) specific subpopulations, respectively. (B, D, F, J, N, R) Gene and pathway enrichment analysis was applied to the identified PPI networks. Based on their log10(P) values, the best 3 scoring biological meanings were illustrated in bar charts for the PDL- (B, F, N) and gingiva- (D, J, R) specific MSC subpopulation. (H, L, P, T) Gene and pathway enrichment analysis of each MCODE individually deciphered the biological meanings of each MCODE network. Based on their log10(P) values, the top 3 scored enriched ontology terms were illustrated in bar charts for each MCODE in the PDL- (H) and gingiva- (L) specific MSCs subpopulations of donor 3. In donor 5, the top 3 scored enriched ontology terms were illustrated in bar charts for MCODE 1–3 and MCODE 1, 2, and 4 in the PDL- (P) and gingiva- (T) specific MSCs subpopulations, respectively. No significantly enriched ontology terms were identified for the remaining MCODEs in either subpopulation of donor 5. The bar charts are colored depending on the corresponding MCODE.

Pathway and process enrichment analysis was additionally performed on identified PPI networks within the upregulated genes of the PDL- and gingiva-derived subpopulations. In donor 2, the PPI network of the PDL-derived subpopulation was related to the transcriptional regulation of white adipocyte differentiation (Fig. 3A–B). The response to corticotropin-releasing hormone and the nuclear receptor transcription pathway were enriched in the PPI network of the gingiva-derived subpopulation (Fig. 3C–D). No densely connected protein networks were detected.

In donor 3, the PPI network of the PDL-derived subpopulation was related to extracellular matrix (ECM) organization, and angiogenesis (Fig. 3E–F). We identified 1 densely connected protein network (MCODE 1) (Fig. 3G–H). Hemostasis and response to inorganic substances were enriched in the protein network of the gingiva-derived subpopulation (Fig. 3I–J). Three MCODEs with different significantly enriched ontology terms were identified (Fig. 3K–L).

In donor 5, the network of the PDL-derived subpopulation was related to actin filament-based processes and Rho GTPases signaling (Fig. 3M–N). Six different densely connected networks were identified, but three did not contain significantly enriched ontology terms (Fig. 3O–P). In the gingiva-derived subpopulation, the PPI network and MCODE 1 were associated with the integrin 1 pathway, ECM organization, and structural components of basement membranes (Fig. 3Q–R). Three further MCODEs were identified, but only two showed significantly enriched terms (Fig. 3S–T).

Differences in the gene signatures, the enriched ontology terms, and PPI networks between tissue-specific and mixed MSC subsets are described in the supplemental appendix (Appendix Fig. 14 to 23 and extended results version).

### Exploring Donor-to-Donor Heterogeneity of Tissue-Specific MSC Subpopulations

**Figure 4.**
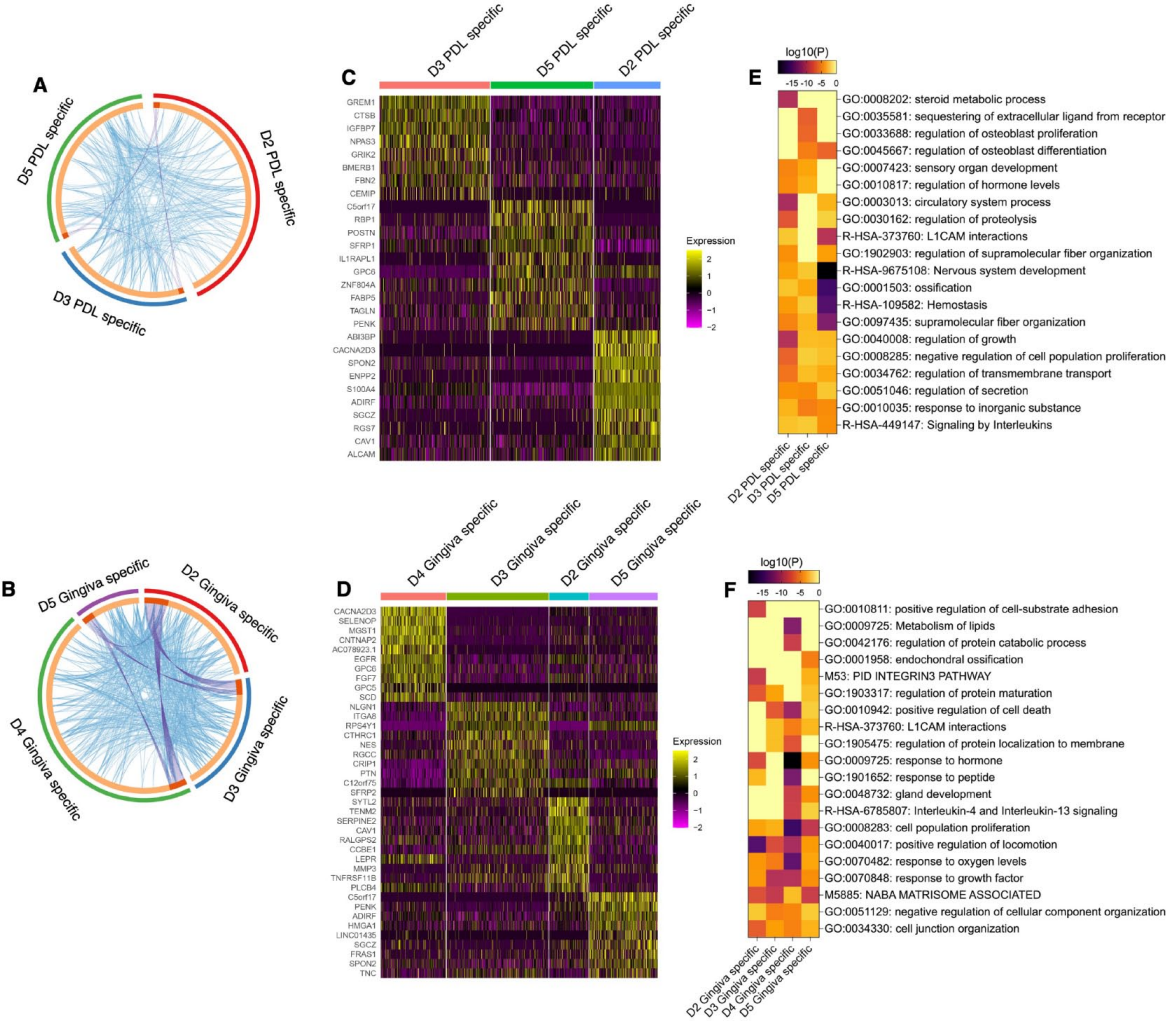
Differential gene expression (DGE) and gene/pathway enrichment analysis comparing tissue-specific subpopulations between donors. (**A, B**) Circos plot illustrates the overlap of significantly upregulated genes of the periodontal ligament (PDL)– (A) or gingiva- (B) specific subpopulations between different donors. The light orange arc per subpopulation depicts the uniqueness of the genes in each subpopulation. Genes that belong to at least 2 different subpopulations are illustrated in red and are linked by purple lines. The blue lines converge genes between different subpopulations that share the same significantly enriched ontology terms. The red, blue, green, and violet arcs present the significantly upregulated genes in the tissue-specific subpopulations of the different donors. (**C, D**) Gene expression heat map depicting the top 10 differently expressed genes in individual cells of the PDL- (C) or gingiva- (D) specific subpopulations between donors. **(E, F)** Enriched terms in the PDL- (E) and gingiva- (F) specific subpopulations compared between donors are displayed by heat maps. Each term represents a cluster of significantly enriched pathways and/or 10.

We examined the gene signatures of tissue-specific MSCs subpopulations between different donors. Comparing the gene expression of the PDL- or gingiva-specific subpopulations identified significantly upregulated genes in donors 2, 3, and 5 (Fig. 4A) and in donors 2, 3, 4, and 5 (Fig. 4B), respectively. Overlaps of upregulated genes between the 3 PDL-specific (Fig. 4A) and 4 gingiva-specific (Fig. 4B) subpopulations were detected.

DGE analysis revealed donor-specific gene signatures for both tissue-specific subpopulations within the top 10 differently expressed genes. These gene signatures are depicted in heat maps (Fig. 4C, D) and are described in the supplemental appendix (extended results version).

Many identified gene ontology clusters within the significantly upregulated genes contained terms enriched in more than 1 donor. Only a few gene ontology clusters among the top 20 clusters were exclusively enriched in 1 donor (Fig. 4E–F and Appendix Fig. 12–13).

In the PDL-derived subpopulations, donors 2 and 5 contained 1 specific enriched cluster, and donor 3 had 2 specific enriched clusters. Donor 2 was related to steroid metabolic processes. Donor 3 was associated with the sequestering of extracellular ligands from receptors and the regulation of osteoblast proliferation. Genes exclusively enriched in the PDL-specific subpopulation of donor 5 were associated with RHO GTPases signaling, gap junction trafficking, and regulation (Fig. 4E and Appendix Fig. 12).

Within the gingiva-derived subpopulations, donors 2 and 4 exhibited 1 and 2 exclusively enriched clusters, respectively. Donor 2 was enriched in the regulation of cell-substrate adhesion. The gingiva-specific subpopulation of donor 4 contained genes involved in protein catabolic processes and lipids metabolism (Fig. 4F and Appendix Fig. 13). Together, these results suggest a limited functional variability in the tissue-specific MSC subpopulations between different donors.

**Figure 5.**
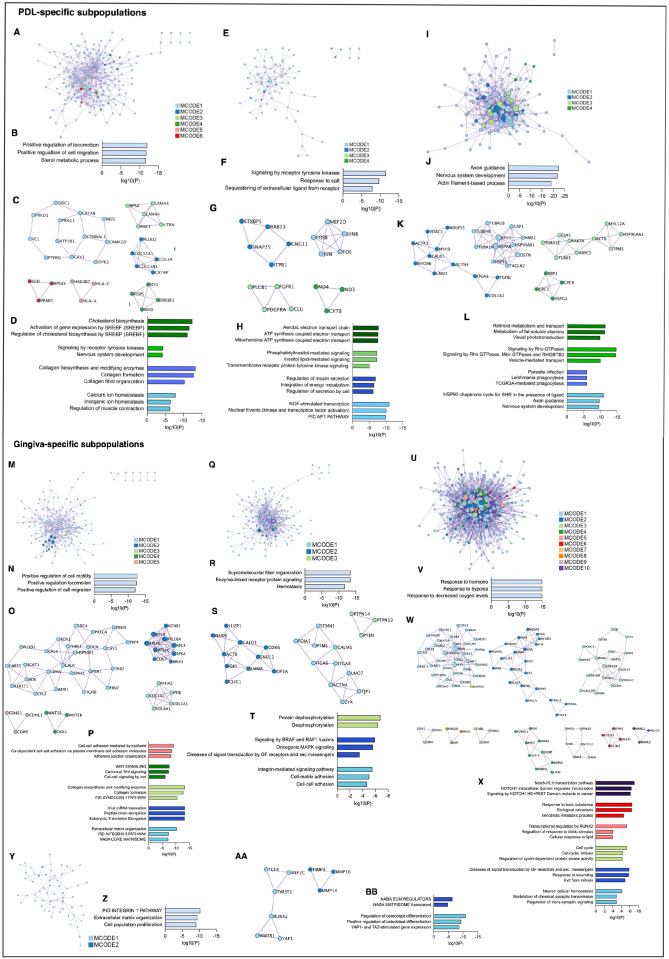
Protein-protein interaction (PPI) and Molecular Complex Detection (MCODE) identification within upregulated genes of periodontal ligament (PDL)– (A–L) and gingiva- (M–BB) specific subpopulations in donors 2, 3, 4, and 5. (A, E, I, M, Q, U, Y) Upregulated PPI networks identified within the PDL- and gingiva-specific subpopulations of donors 2 (A, M), 3 (E, Q), 4 (U), and 5 (I, Y). (C, G, K) Running the MCODE algorithm on the PPI networks identified 6 (MCODE 1–6), 4 (MCODE 1–4), and 4 (MCODE 1–4) densely connected protein networks in the PDL-specific subpopulations of donors 2 (C), 3 (G), and 5 (K), respectively. (O, S, W, AA) In the gingiva-specific subpopulations of donors 2 (O), 3 (S), 4 (W), and 5 (AA), 5 (MCODE 1–5), 3 (MCODE 1–3), 10 (MCODE 1–10), and 2 (MCODE 1–2) were identified, respectively. (B, F, J, N, R, V, Z) Gene and pathway enrichment analysis was applied to all PPI networks. Based on their log10(P) values, the top 3 scored biological meanings were illustrated in bar charts for the PDL- and gingiva-specific mesenchymal stromal cell (MSC) subpopulations of donors 2 (B, N), 3 (F, R), 4 (V), and 5 (J, Z). (D, H, L, P, T, X, BB) Gene and pathway enrichment analysis of each MCODE individually deciphered the biological meanings of each MCODE network. Based on their log10(P) values, the top 3 scored enriched ontology terms were illustrated in bar charts for MCODE 1–4, MCODE 1–4, and MCODE 1–4 in the PDL-specific MSC subpopulations of donors 2 (D), 3 (H), and 5 (L), respectively. No significantly enriched ontology terms were identified for MCODE 5 and 6 in the PDL-specific subpopulation of donor 2 (C, D). The top 3 scored enriched ontology terms were illustrated in bar charts for MCODE 1–5; MCODE 1–3; MCODE 1–3, 5, 6, and 10; and MCODE 1–2 in the gingiva-specific MSCs subpopulations of donors 2 (P), 3 (T), 4 (X), and 5 (BB), respectively. No significantly enriched ontology terms were identified for MCODE 4 and 7–9 in the gingiva-specific MSC subpopulations of donor 4 (W, X). The bar charts are colored depending on the corresponding MCODE.

Pathway and process enrichment analyses were performed on the identified PPI networks within the significantly upregulated genes. In the PDL-specific subpopulation of donor 2, the PPI network was related to the regulation of cell migration and the sterol metabolic process (Fig. 5A–B). Four of the 6 densely connected protein networks (MCODE) were associated with various processes (Fig. 5C–D). Signaling by receptor tyrosine kinase, response to salt, and sequestering of extracellular ligands from receptors were enriched in the PPI network of donor 3 (Fig. 5E–F), and 4 densely connected protein networks were identified (Fig. 5G–H). The PPI network in donor 5 was linked to axon guidance, nervous system development, and actin filament-based processes (Fig. 5I–J). Axon guidance and nervous system development were also related to MCODE 1. Three further MCODEs enriched with different terms were identified (Fig. 5K–L).

In the gingiva-specific subpopulation of donor 2, the PPI network was related to the regulation of cell motility and migration (Fig. 5M–N). Five different MCODEs were found in this donor (Fig. 5O–P). The PPI network in donor 3 was linked to supramolecular fiber organization, enzyme-linked receptor protein signaling, and hemostasis (Fig. 5Q–R). In this donor, three MCODEs enriched with functional terms were identified (Fig. 5S–T). In donor 4, the PPI network of the gingiva-specific subpopulation was involved in the response to hormones and hypoxia (Fig. 5U–V). Ten different MCODEs were identified, but only 6 of them contained significantly enriched ontology terms (Fig. 5W–X). In donor 5, the PPI network was related to the integrin 1 pathway, ECM organization, and cell proliferation (Fig. 5Y–Z). Two MCODEs enriched with ontology terms were found (Fig. 5AA–BB).

## Discussion

The easy accessibility of PDL and gingiva makes PDL- and gingiva-derived MSCs an alternative to less accessible MSCs. Preclinical and clinical studies have already investigated their potential to treat diseases, including periodontal diseases and gingival recessions (Yamada et al. 2020). Studies have already attempted to compare PDL- and gingiva-derived MSCs, demonstrating some differences in their properties (Xing et al. 2019; Abedian et al. 2020), without considering the cellular heterogeneity of MSCs. This study is the first using single-cell transcriptome analysis to understand the tissue-to-tissue and the variability between donors of in vitro cultured PDL- and gingiva-derived MSCs. A considerable advantage of this design is that the isolation of PDL- and gingiva-derived MSCs from the same individuals and the clustering in donor- or tissue-specific ways allows us to investigate exclusively tissue-to-tissue variability by excluding donor-to-donor variability and vice versa.

The same sampling method and culture conditions were used for all used MSCs to minimize the influence of the artificial niche in culture. In addition, MSCs with similar passages were used. We decided to examine in vitro cultured MSCs since MSCs must be cultured, expanded, and sometimes treated or modified ex vivo before the transplantation (Levy et al. 2020). Hence, the observed single-cell transcriptomes of in vitro cultured MSCs may better reflect the situation in transplanted MSCs than in situ single-cell transcriptomes.

This study identified PDL- and gingiva-specific MSC subpopulations with unique gene expression signatures. This was the first time gingiva-derived MSC subpopulations were detected. A recent study (Xu et al. 2023) already demonstrated PDL-derived MSC subpopulations when comparing PDL- and dental pulp–derived MSCs. However, in this work, the PDL and dental pulp were obtained from different donors and could not be considered “pure” tissue specific.

The PDL- and gingiva-derived MSC subpopulations showed several functional overlaps and heterogeneity restricted to specific functional processes. The regulation of adipocyte differentiation, fibroblast proliferation, cell-substrate adhesion, and ECM-associated processes were exclusively enriched in the PDL-derived subpopulations. In contrast, developmental processes were solely associated with the gingiva-derived subpopulations. These differences are in accordance with functional studies, showing differences in biological properties, including the proliferation (Santamaría et al. 2017; Xing et al. 2019; Abedian et al. 2020) of PDL- and gingiva-derived MSCs. In addition, the enriched ECM-associated processes mirror the PDL’s nature of specialized connective tissue (Beertsen et al. 1997). This tissue-of-origin heterogeneity is in line with previous scRNA-seq studies, showing tissue-to-tissue differences between PDL- and dental pulp–derived MSCs (Pagella et al. 2021; Lee et al. 2022; Xu et al. 2023). PPI network and MCODE analysis confirmed the tissue-of-origin heterogeneity, although ECM-associated and cell-ECM interaction processes were detected in both PDL- and gingiva-derived subpopulations.

This functional analysis additionally points to donor-to-donor differences showing donor-dependent different grades of functional overlaps between PDL- and gingiva-derived subpopulations, differences in the tissue-specific enriched biological functions/processes between the investigated donors, and donor-dependent enrichment of multiple processes that were found in both tissue-derived subpopulations.

Despite the presence of some tissue-specific subpopulations, the heterogeneity found between gingiva- and PDL-derived MSCs was rather limited: tissue-specific subpopulations were found in only 3 of the 5 investigated donors. Furthermore, the differences between gingiva- and PDL-specific subpopulations were discordant between donors. This fact raises the question of how far gingiva- and PDL-derived MSCs differ (Palmer and Lubbock 1995). On one hand, large differences in the properties of gingiva and PDL MSCs are hard to expect because both tissues originate predominantly from the neural crest (Coura et al. 2008; Xu et al. 2013). On the other hand, the tissue-dependent MSCs’ niche diversity and different environmental factors might contribute to MSCs’ heterogeneity. The MSC heterogeneity additionally depends on several factors, including donor, tissue source, anatomical location in the tissue, and culture conditions of the artificial niche (Costa et al. 2021). Therefore, we have standardized the culture conditions. Well-controlled culture conditions might drive the selection of certain MSC subpopulations and thus might have contributed to the limited inter-tissue variability.

Gene expression analysis of PDL- or gingiva-derived MSC subpopulations across donors identified differently expressed genes. However, multiple significantly upregulated genes were found in more than one donor, indicating a confined donor-to-donor variability of the tissue-derived MSC subpopulations. This was confirmed by pathway/process enrichment analysis, showing a great functional overlap of the tissue-derived subpopulations between the different donors. Nevertheless, exclusively enriched functional processes were detected in the PDL- or gingiva-derived subpopulations.

Besides tissue-derived MSC subpopulations, different numbers of mixed subsets were detected depending on the donor. This indicates that isolated MSC populations also contain cells with a gene expression profile independent of the tissue origin. This further suggests a certain degree of intercellular heterogeneity when comparing them with tissue-specific subsets within a given MSC population. This would coincide with previous studies demonstrating that MSCs are diverse within a population (Costa et al. 2021).

The existence of tissue- and donor-specific MSC subpopulations might affect their therapeutic potential, but we are still too far from understanding this relationship. The mechanisms involved in the clinical effectiveness of MSCs are not yet understood: MSCs usually do not differentiate after transplantation and usually stimulate endogenous regenerative mechanisms through the secretion of trophic factors and immunomodulators (Wang et al. 2014). Selecting some subpopulations with favorable properties and using them for clinical purposes looks attractive. However, this can hardly be done using flow sorting or magnetic separation because these subpopulations need to have some specific surface markers. However, the existence of such a marker is questionable despite functional heterogeneity. A more promising approach would be variating the culture conditions, but we still need to understand their impact on the gene expression profiles.

In conclusion, this study gives insights into the cellular composition of PDL- and gingiva-derived MSCs populations and their heterogeneity, which will help develop procedures to reduce heterogeneity and increase their therapeutic efficiency. It highlights the tissue- and donor-specific heterogeneity in the transcriptomes of PDL- and gingiva-derived MSCs. Identifying tissue-specific and mixed subsets demonstrates the intercellular differences in PDL- and gingiva-derived MSCs populations. This study first detected a gingiva-specific MSC subpopulation. Although the different MSC subpopulations demonstrated specific gene expression profiles, several overlaps were found on the gene and functional level, indicating a restriction of heterogeneity to specific cellular processes. Nevertheless, it becomes increasingly clear that the tissue, donor source, and cellular composition of a given MSCs population should be considered in the quality check before ex vivo expanded MSCs are transplanted.

## Author Contributions

C. Behm, contributed to conception and design, data acquisition, analysis, and interpretation, drafted and critically revised the manuscript; O. Miłek, contributed to conception, data acquisition and analysis, drafted and critically revised the manuscript; K. Schwarz, contributed to data analysis, critically revised the manuscript; A. Kovar, X. Rausch-Fan, A. Moritz, contributed to data interpretation, critically revised the manuscript; S. Derdak, contributed to data acquisition and analysis, drafted and critically revised manuscript; O. Andrukhov, contributed to conception, data analysis and interpretation, drafted and critically revised the manuscript. All authors gave their final approval and agreed to be accountable for all aspects of the work.

## Supplemental Material

sj-docx-1-jdr-10.1177_00220345241271997 – Supplemental material for Heterogeneity in Dental Tissue–Derived MSCs Revealed by Single-Cell RNA-seq Supplemental material, sj-docx-1-jdr-10.1177_00220345241271997 for Heterogeneity in Dental Tissue–Derived MSCs Revealed by Single-Cell RNA-seq by C. Behm, O. Miłek, K. Schwarz, A. Kovar, S. Derdak, X. Rausch-Fan, A. Moritz and O. Andrukhov in Journal of Dental Research
