# Supplementary material for: Heterogeneity in Dental Tissue–Derived MSCs Revealed by Single-Cell RNA-seq
Source: J Dent Res. 2024 Sep 26;103(11):1141–52. doi: 10.1177/00220345241271997 (PMC11500480; doi:10.1177/00220345241271997)
Supplement: sj-docx-1-jdr-10.1177_00220345241271997 – Supplemental material for Heterogeneity in Dental Tissue–Derived MSCs Revealed by Single-Cell RNA-seq [file sj-docx-1-jdr-10.1177_00220345241271997.docx]

**Heterogeneity in dental tissue-derived MSCs revealed by single-cell RNA-seq**

Christian Behm^1^, Oliwia Miłek^1^, Katharina Schwarz^1^, Alexander Kovar^1^, Sophia Derdak^2^, Xiaohui Rausch-Fan^3, 4^, Andreas Moritz^3^ and Oleh Andrukhov^1*^

^1^Competence Center for Periodontal Research, University Clinic of Dentistry, Medical University of Vienna, Vienna, Austria, 1090.

^2^Core Facilities, Medical University of Vienna, Vienna, Austria, 1090.

^3^Clinical Division of Conservative Dentistry and Periodontology, University Clinic of Dentistry, Medical University of Vienna, Vienna, Austria, 1090.

^4^Center for Clinical Research, University Clinic of Dentistry, Medical University of Vienna, Vienna, Austria, 1090.

**Results**


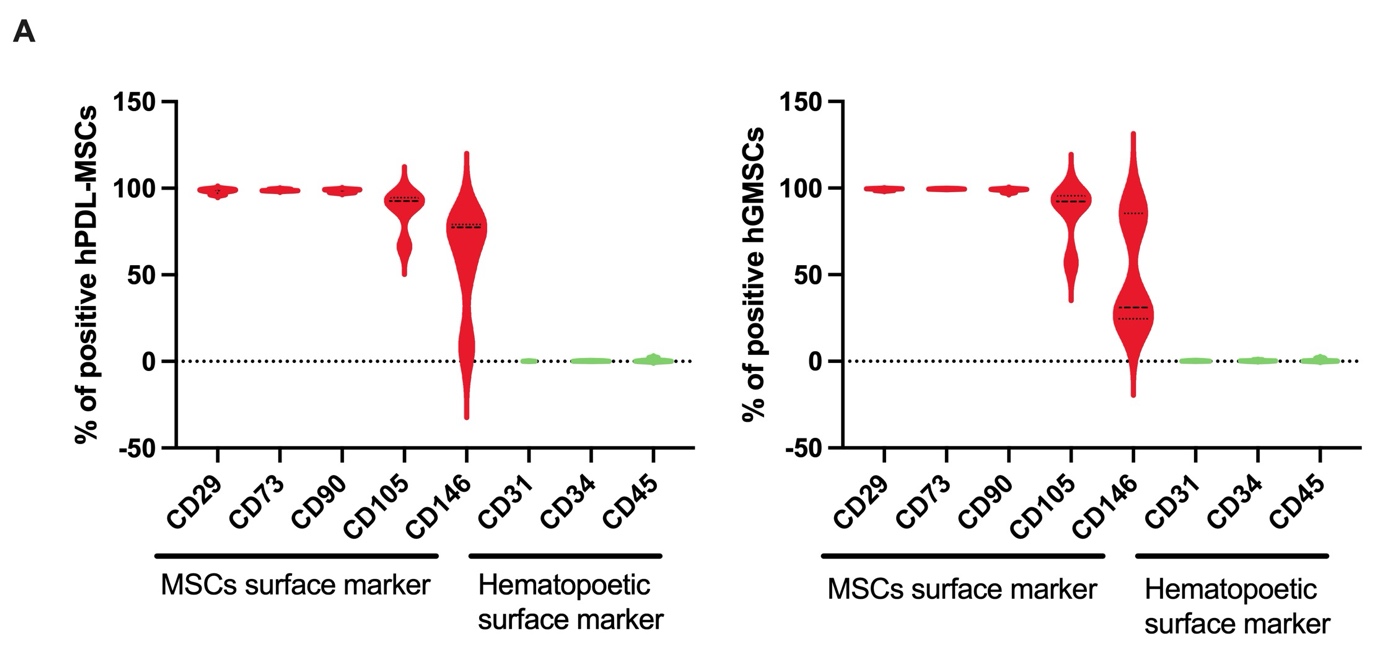


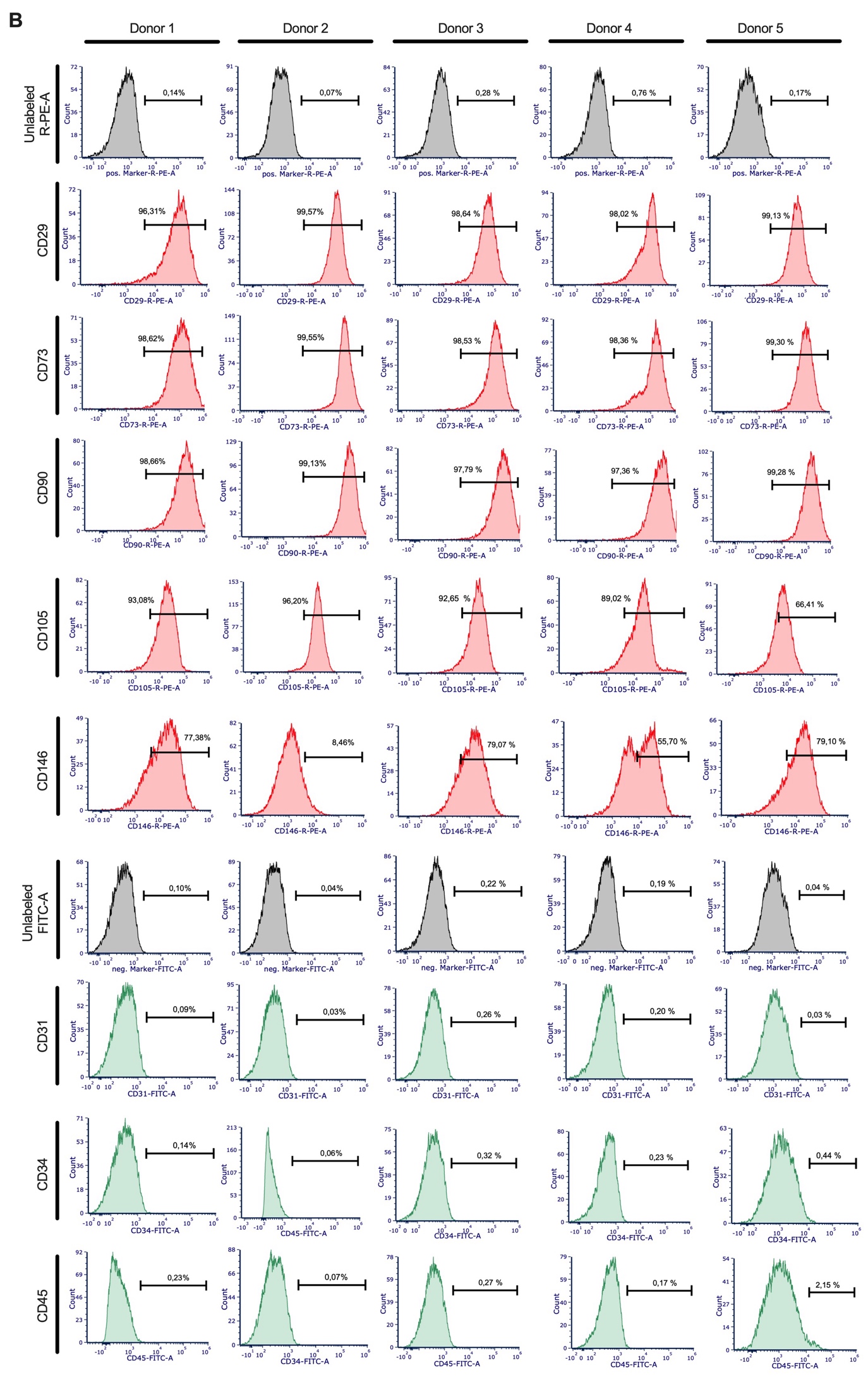


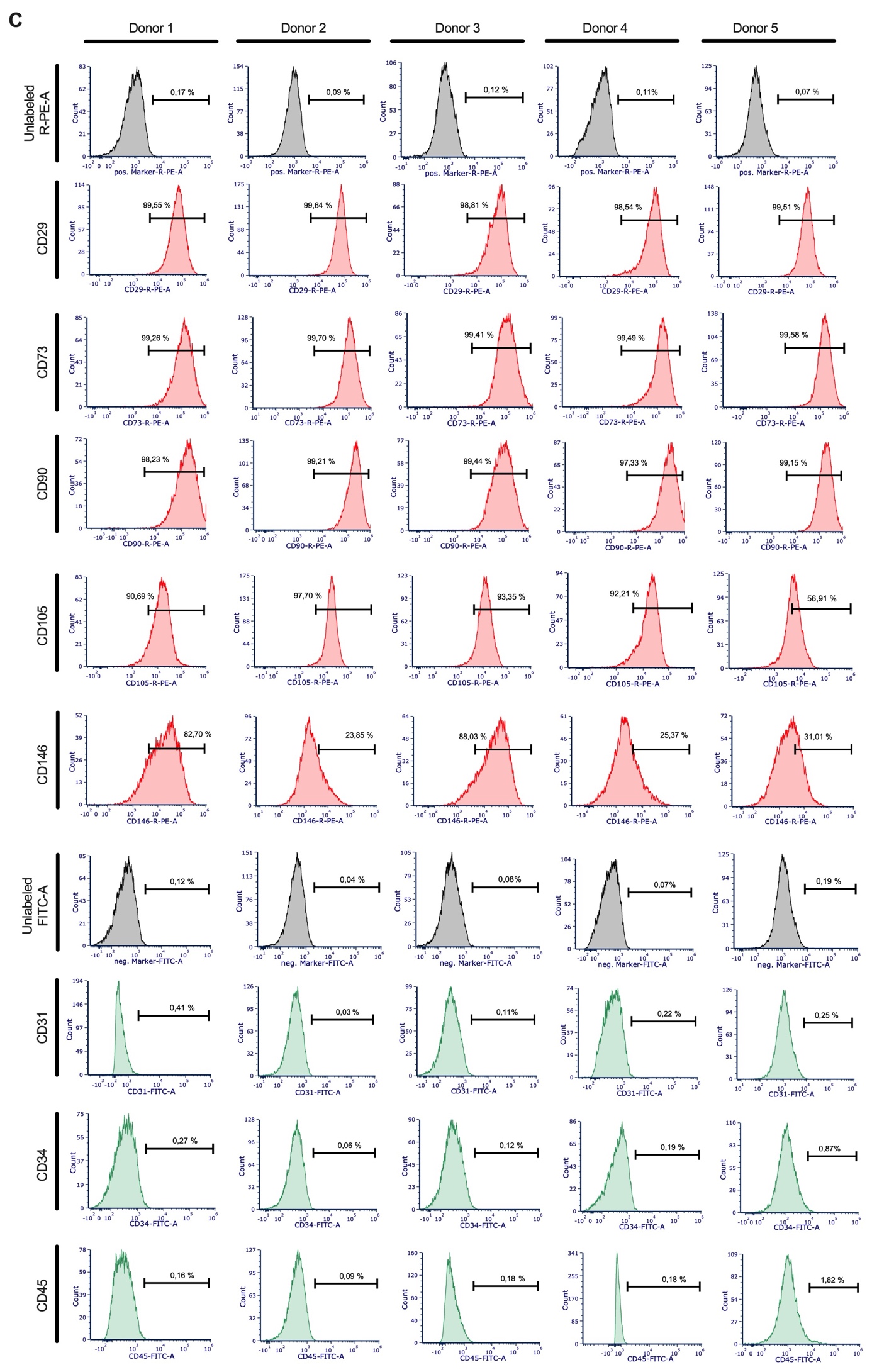


**Appendix Figure 1: Characterization of PDL- and gingiva-derived MSCs by their surface marker expression.** (A) The percentage of CD29, CD73, CD90, CD105, and CD146 (red), and the percentage of CD31, CD34, and CD45 (green) positive PDL- (hPDL-MSCs) and gingiva- (hGMSCs) derived MSCs (n = 5 donors/tissue). The data are presented as violin plots. (B-C) Flow cytometry histograms for all investigated surface markers in PDL- (B) and gingiva- (C) derived MSCs, showing the percentage of positive MSCs for all five donors. MSCs and hematopoietic surface markers are labeled red and green, respectively. Histograms of unlabeled controls are colored in grey.


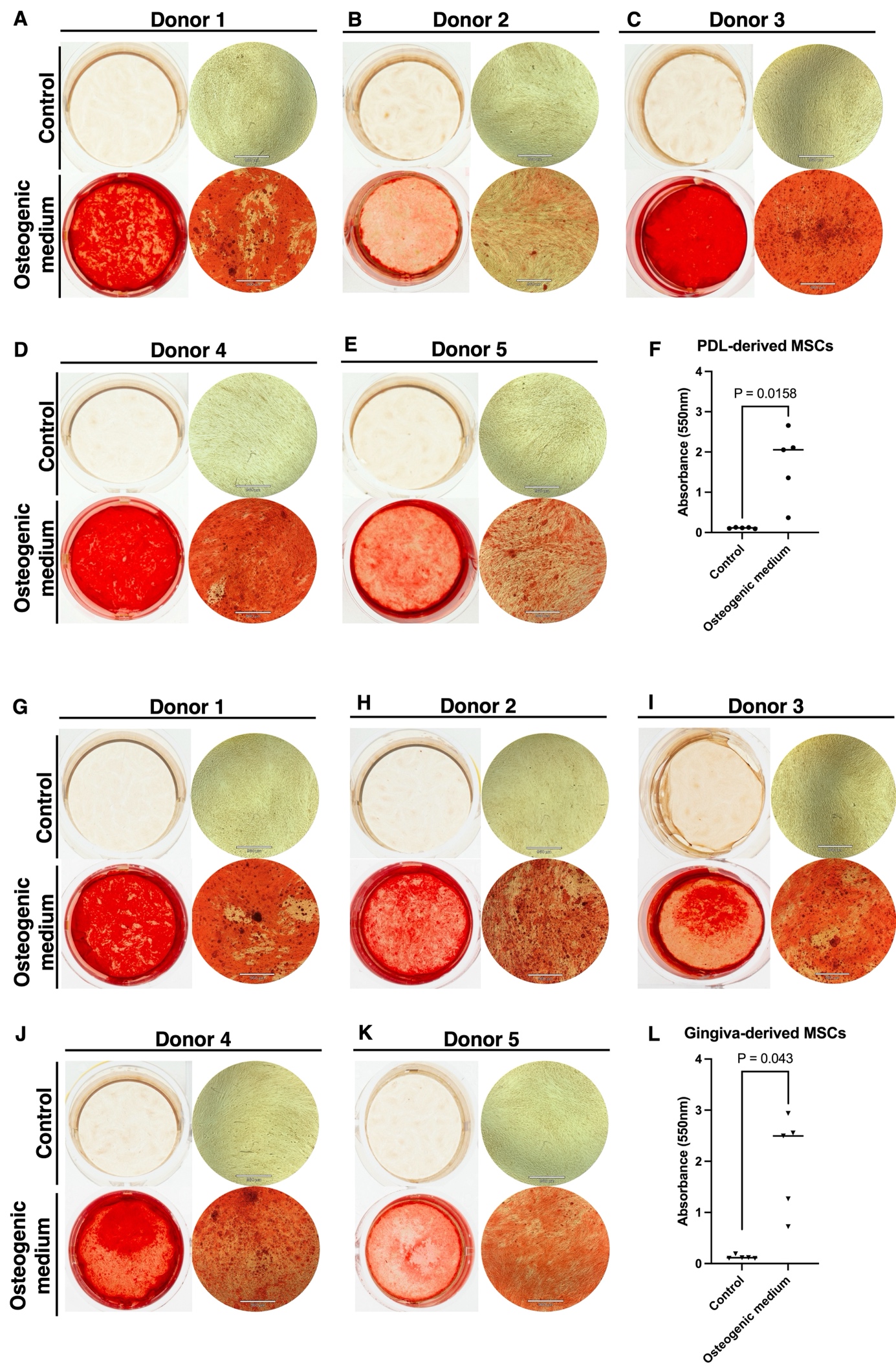


**Appendix Figure. 2: Osteogenic differentiation potential of PDL- and gingiva-derived MSCs isolated from donor 1-5.**

(A-E and G-K) Images from the alizarin red stained PDL- (A-E) and gingiva- (G-K) derived MSCs of donor 1-5. MSCs were cultured in an osteogenic differentiation medium for 21 days. MSCs cultured in DMEM medium served as control. Within each donor panel, pictures from the appropriate whole well and bright-field images are shown on the left-hand and right-hand sides, respectively. Scale bars of the bright-field images: 960µm. (F, L) Photometric quantification of the extracted alizarin red stain in the PDL- (F) and gingiva- (L) derived MSCs (n = 5 / tissue). The data are presented as median and individual points (F) and rectangles (L). Statistically significant P<0.05 (Two-tailed paired t-test (F) and two-tailed Wilcoxon Signed Ranks test for pairwise comparison (L)).


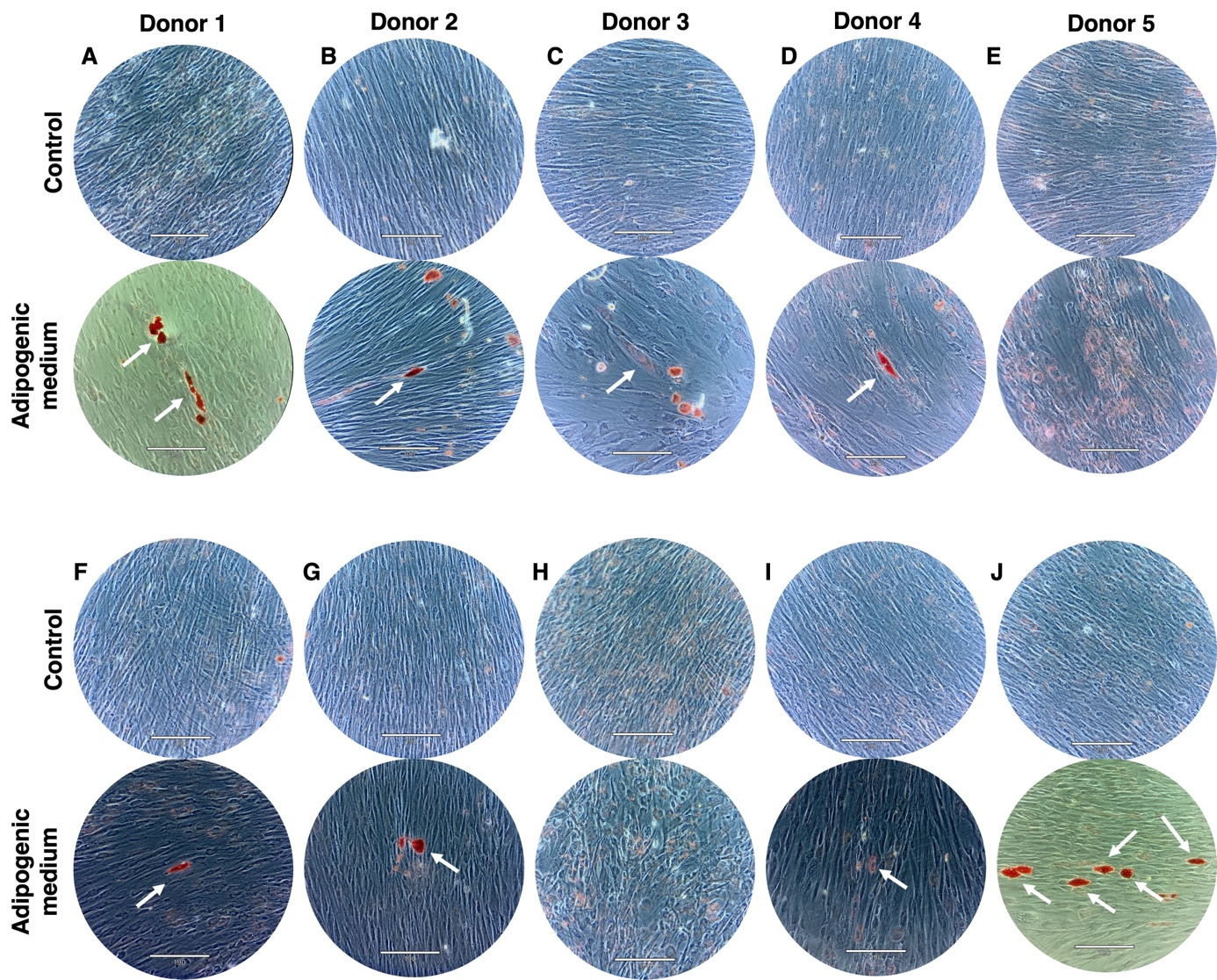


**Appendix Figure. 3: Adipogenic differentiation potential of PDL- and gingiva-derived MSCs isolated from donors 1-5.**

(A-J) Bright-field images from the oil red stained PDL- (A-E) and gingiva- (F-J) derived MSCs of donor 1-5. MSCs were cultured in an adipogenic differentiation medium for 28 days. MSCs cultured in DMEM medium served as control. Scale bars: 190μm.


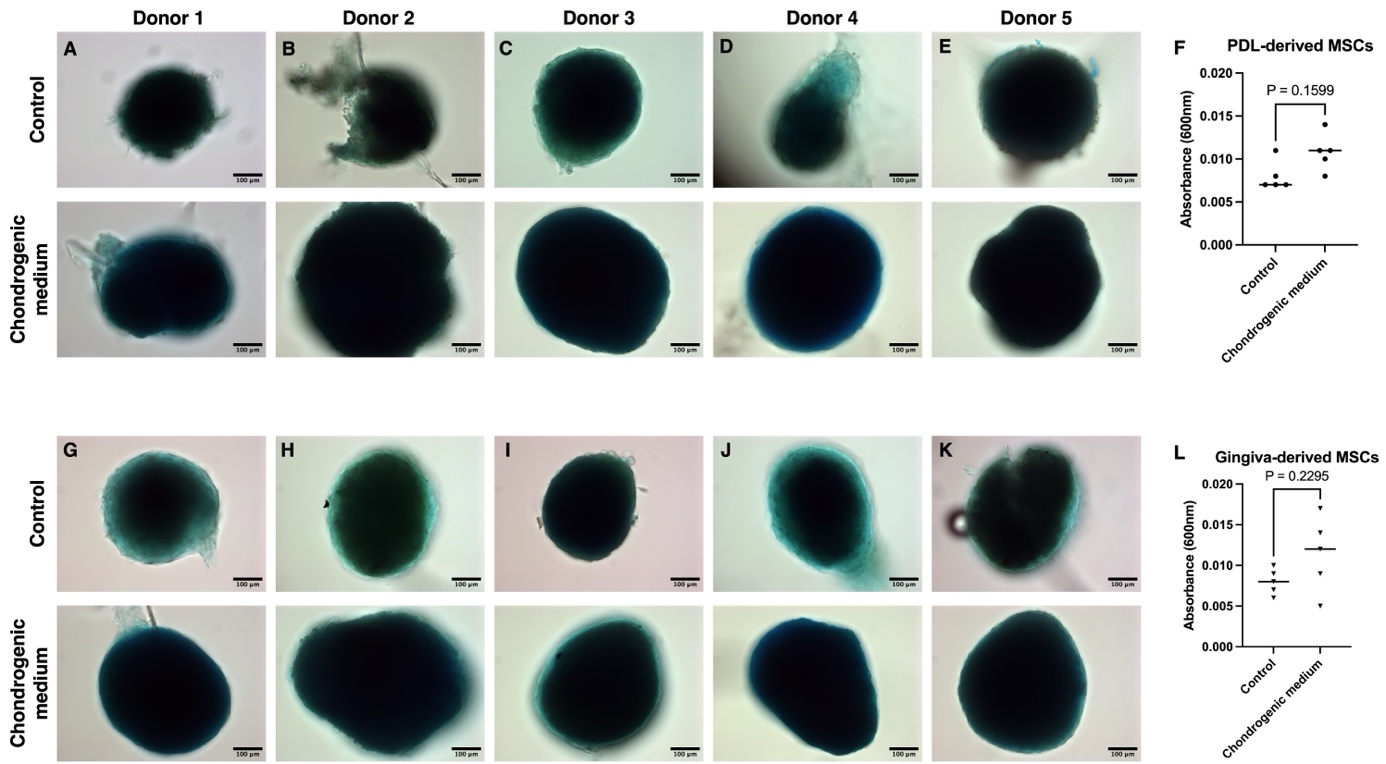


**Appendix Figure. 4: Chondrogenic differentiation potential of PDL- and gingiva-derived MSCs isolated from donor 1-5.**

(A-E, and G-K) Brightfield images from the alcian blue-stained micro-masses built from PDL- (A-E) and gingiva- (G-K) derived MSCs of donor 1-5. MSCs were cultured in a chondrogenic differentiation medium for 21 days. MSCs cultured in DMEM medium served as control. Scale bars: 100μm. (F, L) Photometric quantification of the extracted alcian blue stain in the PDL- (F) and gingiva- (L) derived MSCs (n = 5 / tissue). The data are presented as median and individual points (F) and rectangles (L). No statistical significance (P<0.05) was detected using the Two-tailed paired t-test for pairwise comparison.


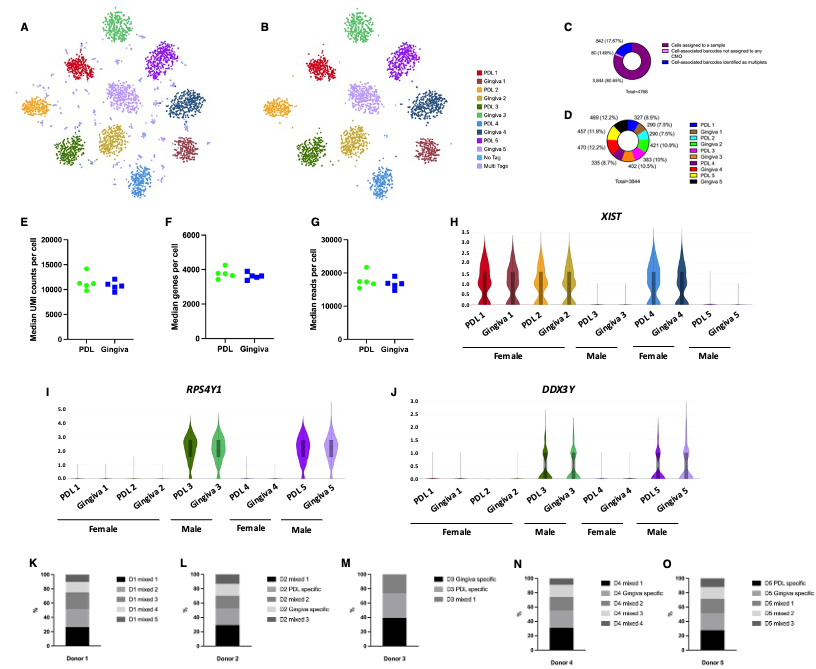


**Appendix Figure. 5: Demultiplexing of hash-tagged scRNA-seq data and quality control metrices.** (A, B) t-SNE projections of demultiplexed data of all 10 samples with (A) and without (B) the exclusion of cell barcodes annotated as “No Tag” and “Multi Tags”. (C) Donut bar shows the percentage of cell barcodes associated with one of the 10 samples, the cell barcodes not associated with any CMO (cell multiplexing oligos), and cell barcodes assigned as multiplets. (D) Distribution of the cell barcodes associated with one of the CMOs (n = 3844) among the 10 different samples. (E, F, G) Quality control metrices showing the median UMI counts per cell (E), the median of detected genes per cell (F), and the median reads per cell (G). The median data per sample are presented as individual data points (n = 5 / tissue type). (h-j) Violin plots demonstrate the gene expression of female (XIST) and male (RPS4Y1 and DDX3Y) specific genes in all 10 samples. The x-axis displays log2-transformed UMI counts. (L-O) The percentage distribution of cells among the different clusters after the re-clustering of PDL- and gingiva-derived MSCs in a donor-specific manner.


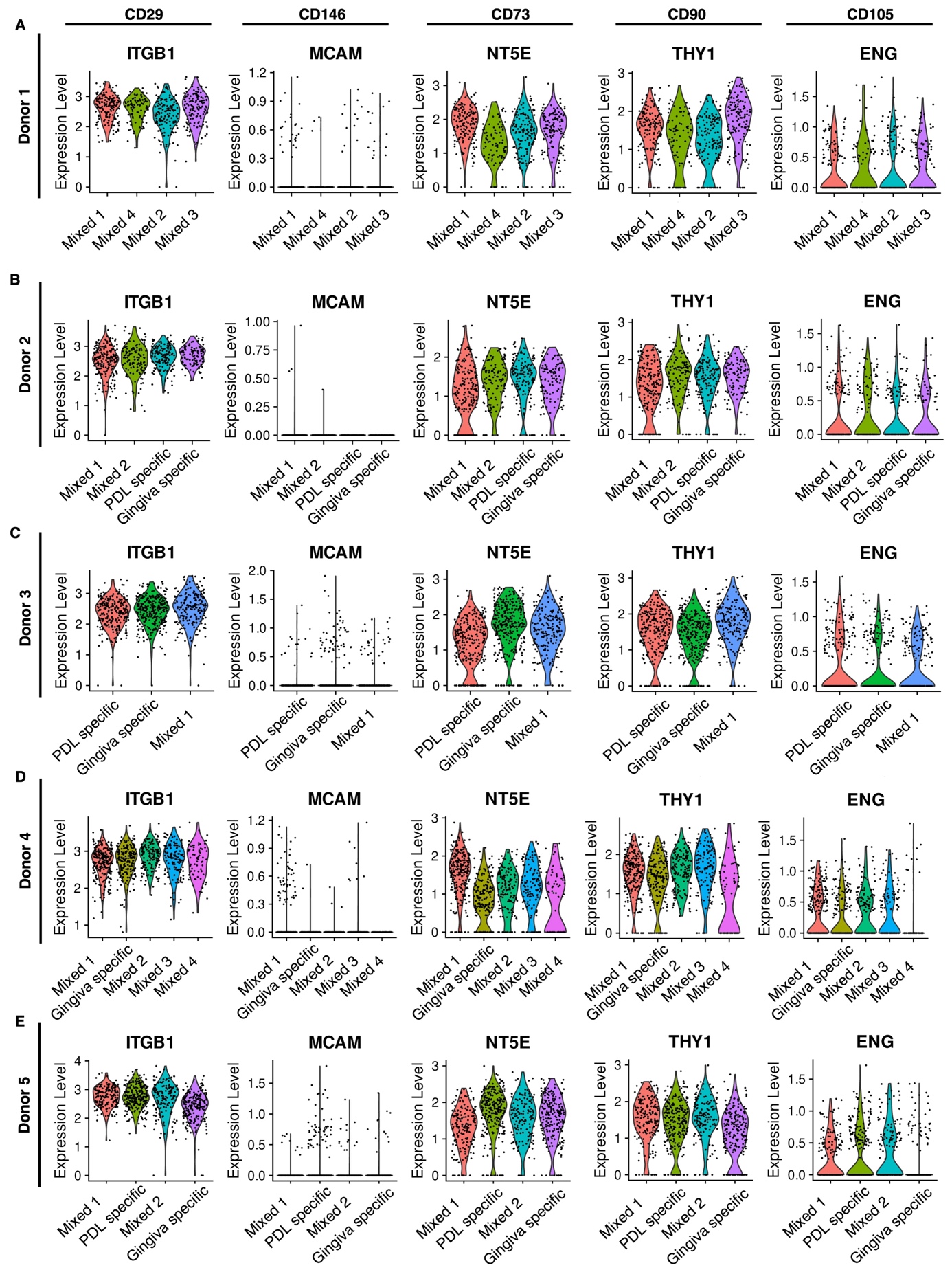
**Appendix Figure. 6: MSCs surface marker gene expression in subpopulations after re-clustering PDL- and gingiva-specific MSCs.** (A-E) Gene expression levels of ITGB1(CD29), MCAM (CD146), NT5E (CD73), THY1 (CD90), and ENG (CD105) in each tissue-specific and mixed subpopulation for donor 1 (A), 2 (B), 3(C), 4 (D), and 5 (E). The data are presented as violin plots and by individual data points for each cell per subpopulation.


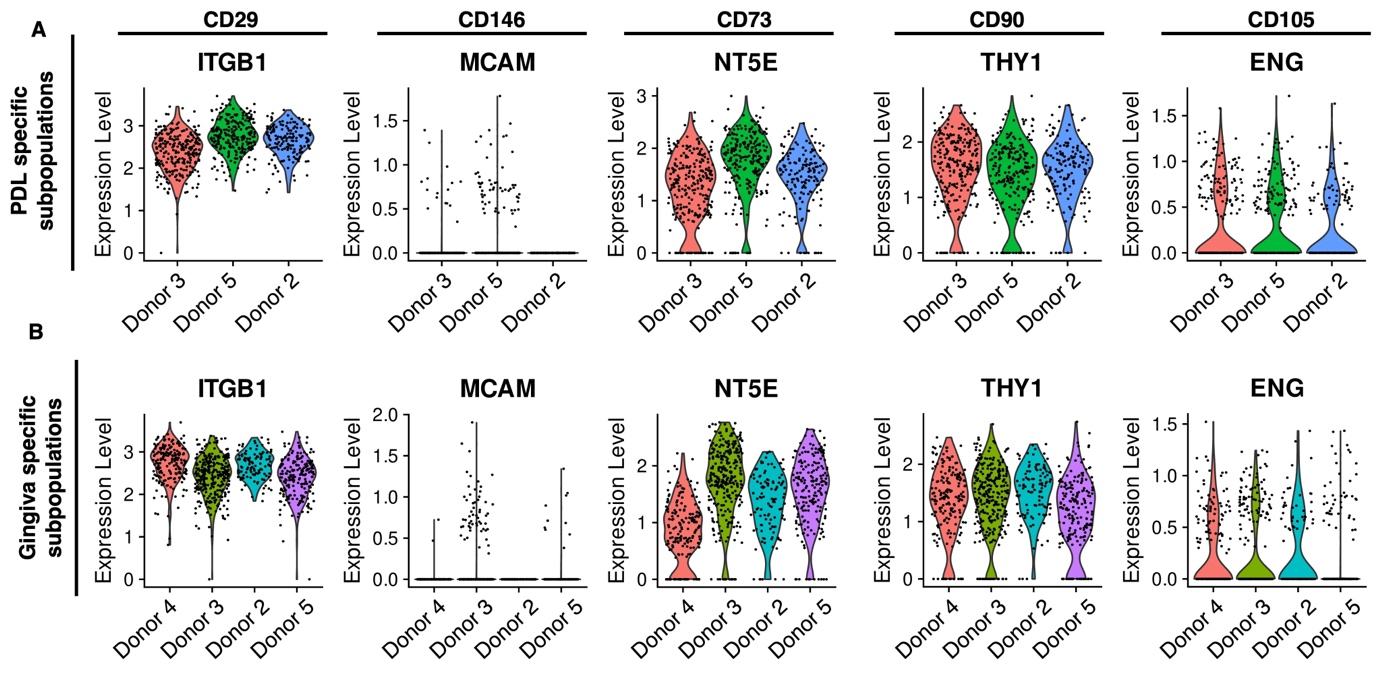


**Appendix Figure. 7: Comparing MSCs surface marker gene expression of PDL- or gingiva-specific subpopulations between different donors.** (A, B) Gene expression levels of ITGB1 (CD29), MCAM (CD146), NT5E (CD73), THY1 (CD90), and ENG (CD105) in PDL- (A) or gingiva- (B) specific subpopulations among donors 3, 5, and 2 or donors 4, 3, 2, and 5, respectively. The data are presented as violin plots and by individual data points for each cell per subpopulation.


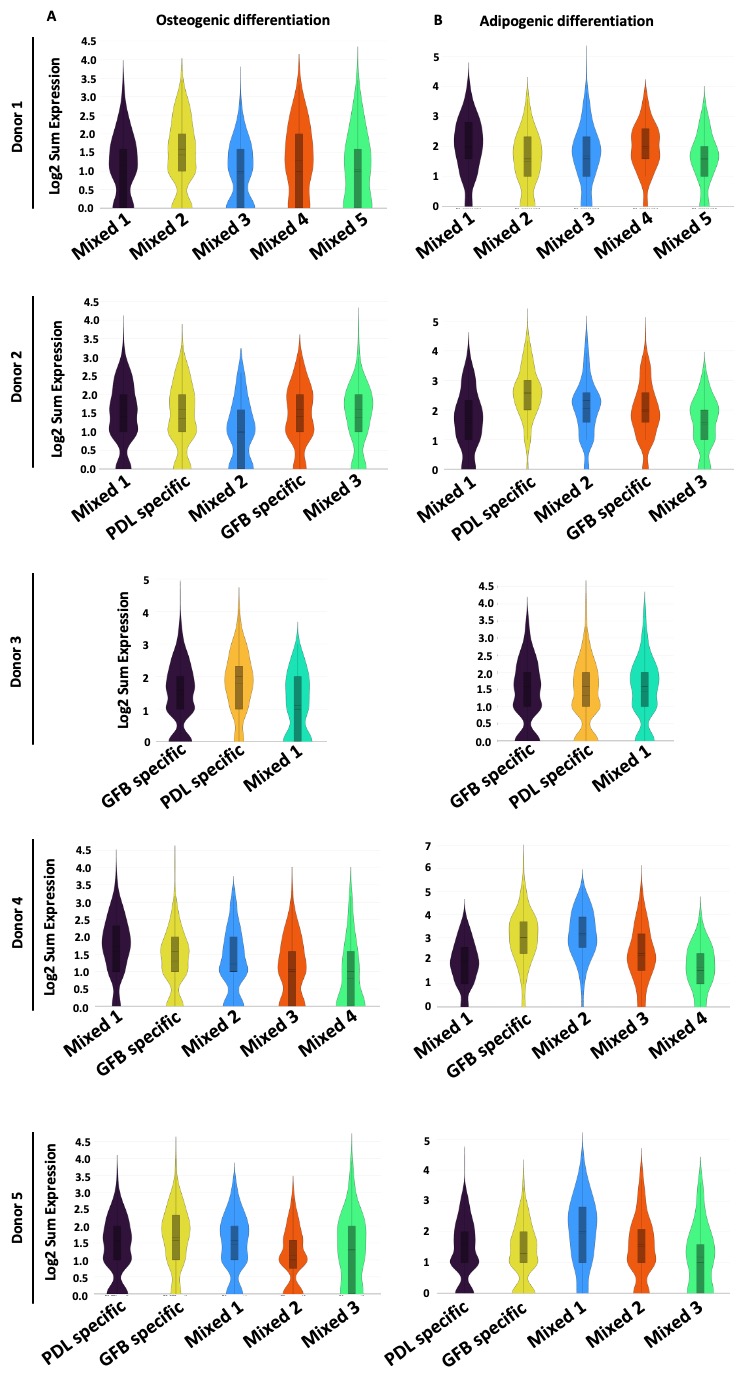


**Appendix Figure. 8: Comparing the gene expression of osteogenic and adipogenic differentiation markers between the detected MSCs subpopulations per donor.** The log2 summed expression of osteogenic (A) and adipogenic (B) differentiation markers is presented as violin plots for each observed MSCs subset. The included osteogenic markers were RUNX2, SP7, BMP2, ALPL, BGLAP, and OPN (A); the included adipogenic markers were PPARG, FASN, SCD, CEBPA, ADIPOQ, and FABP4 (B). The expression of chondrogenic differentiation markers (SOX9, COL2A1, ACAN, and COMP) was very low.


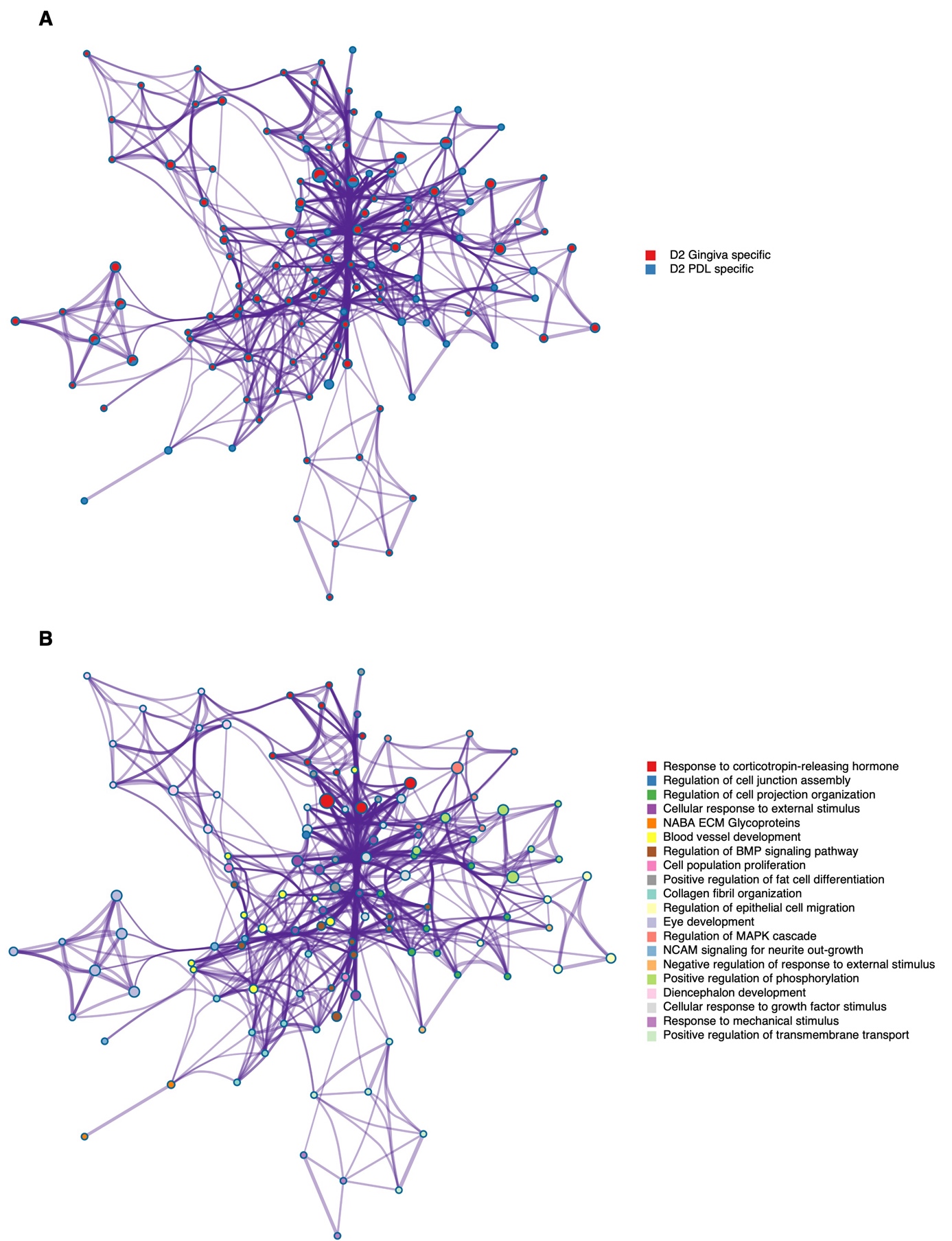
**Appendix Figure. 9: Network of enriched ontology terms found in the tissue-specific MSCs subpopulations of donor 2.** (A, B) Enriched ontology terms were illustrated as an enrichment network, each node representing one specific ontology term. The size of a node is proportional to the total number of genes that fall into this particular ontology term. Enriched terms with a similarity greater than 0.3 are linked by edges, where the thickness of the edges represents the similarity score. In (A), the nodes are presented as pie charts, where the size of the differently colored slices depends on the percentage of genes that belong to the two different tissue-specific MSCs subpopulations in donor 2. In (B), the nodes are colored based on their cluster membership.


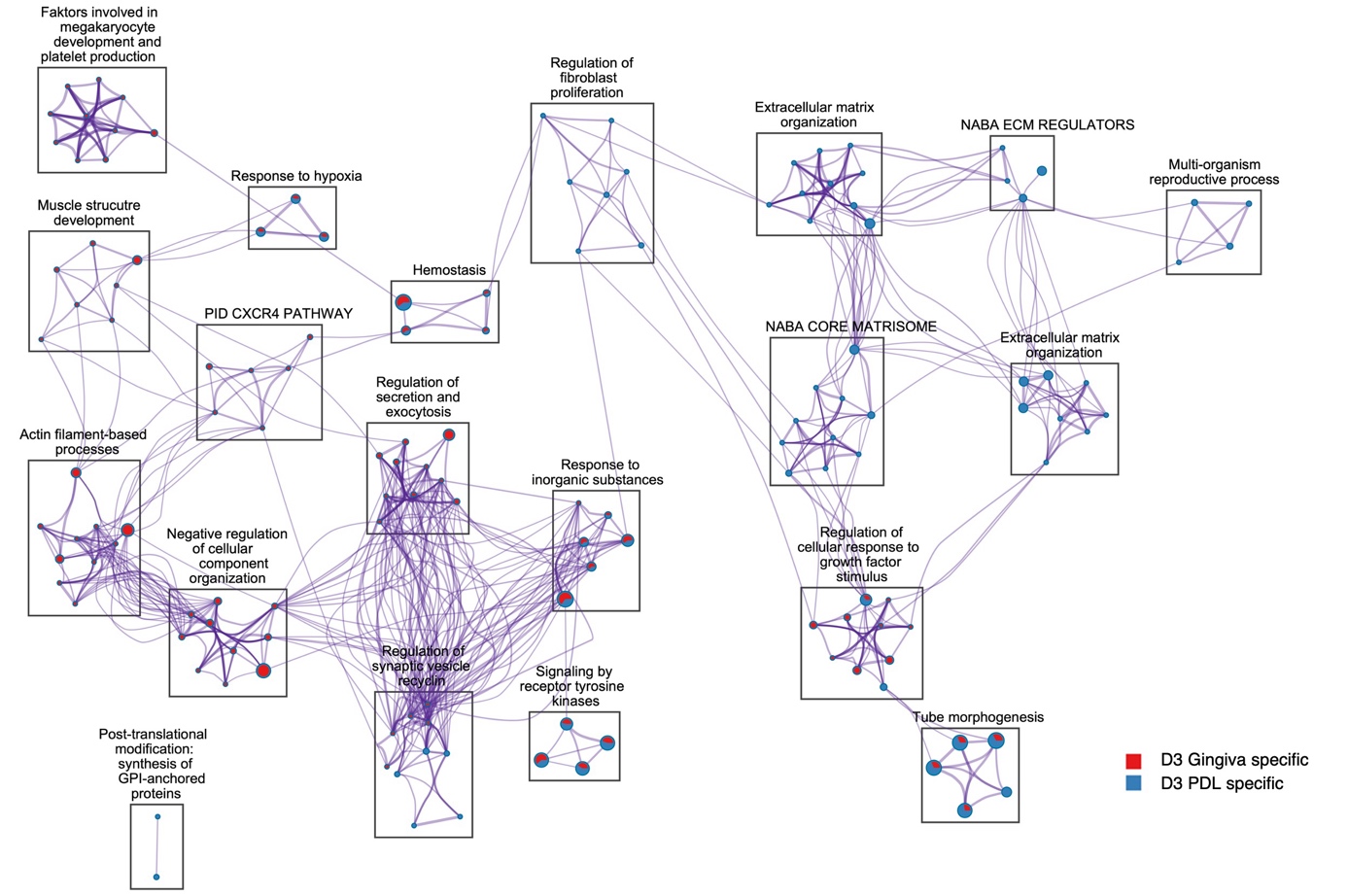
**Appendix Figure 10: Network of enriched ontology terms found in the tissue-specific MSCs subpopulations of donor 3.** Enriched ontology terms are illustrated as an enrichment network in which each node represents one specific ontology term. The size of a node is proportional to the total number of genes that fall into this particular ontology term. Enriched terms with a similarity greater than 0.3 are linked by edges, where the thickness of the edges presents the similarity score. The nodes are shown as pie charts, where the size of the differently colored slices depends on the percentage of genes that belong to the two different tissue-specific MSCs subpopulations in donor 3. Nodes belonging to the same enrichment cluster are grouped and annotated using the best-scored enriched term per cluster as a representative.


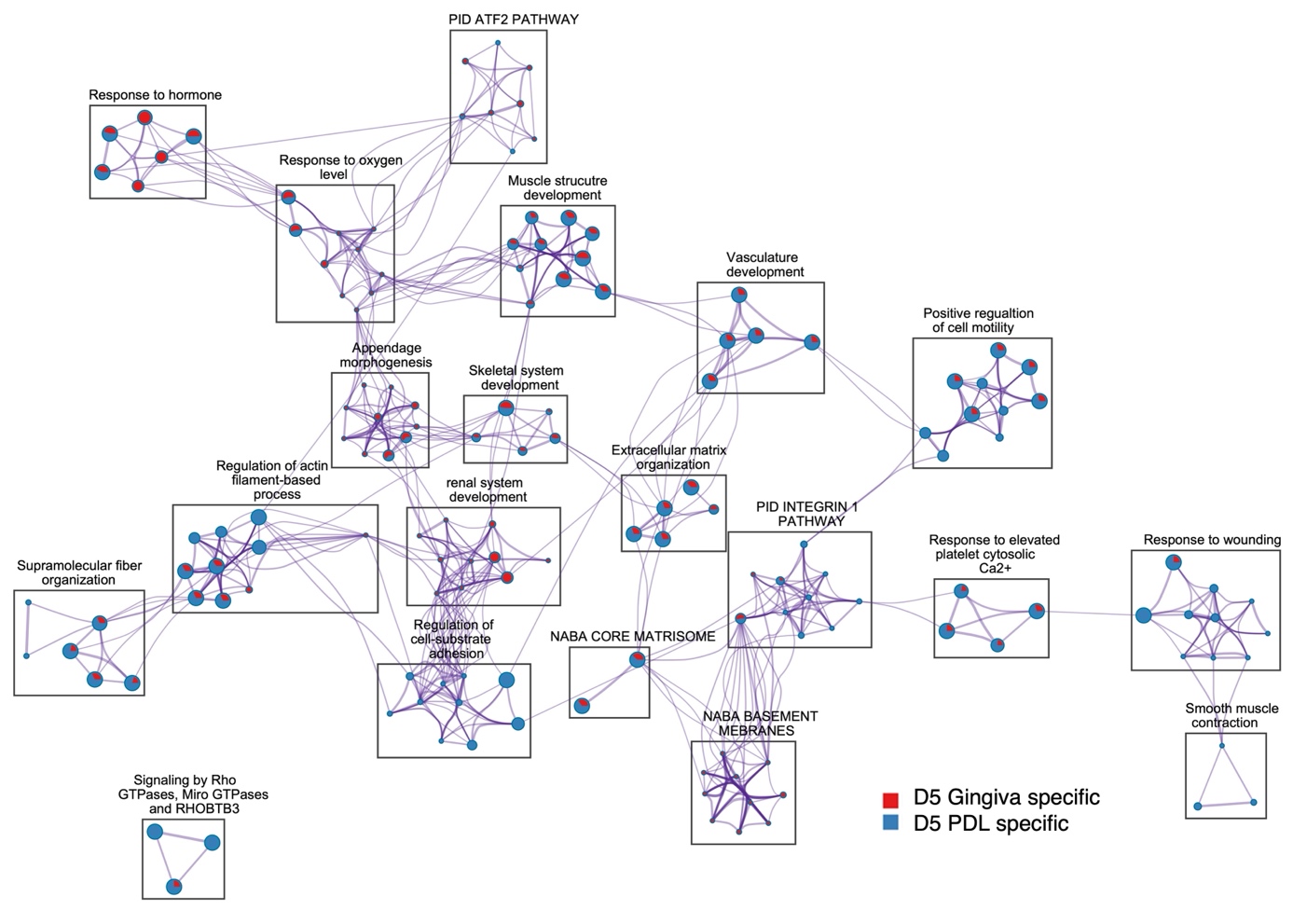
**Appendix Figure. 11: Network of enriched ontology terms found in the tissue-specific MSCs subpopulations of donor 5.** Enriched ontology terms are illustrated as an enrichment network in which each node represents one specific ontology term. The size of a node is proportional to the total number of genes that fall into this particular ontology term. Enriched terms with a similarity greater than 0.3 are linked by edges, where the thickness of the edges presents the similarity score. The nodes are shown as pie charts, where the size of the differently colored slices depends on the percentage of genes that belong to the two different tissue-specific MSCs subpopulations in donor 3. Nodes belonging to the same enrichment cluster are grouped and annotated using the best-scored enriched term per cluster as a representative.


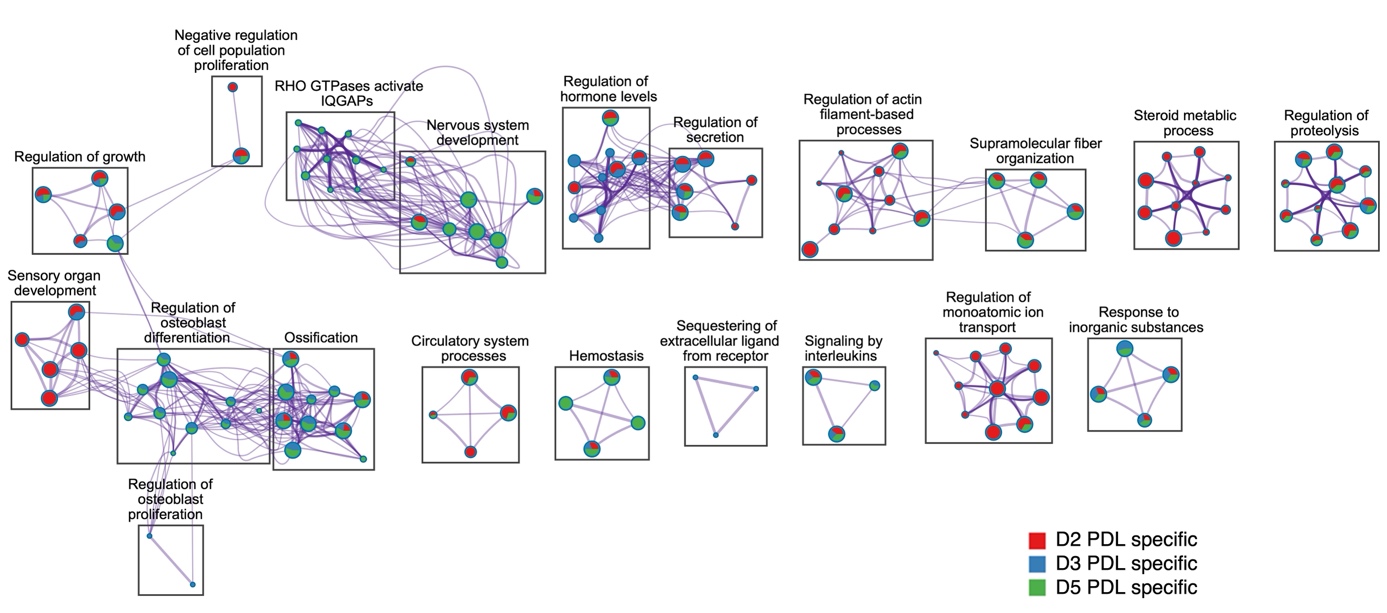
**Appendix Figure 12: Network of enriched ontology terms found in the PDL-specific MSCs subpopulations of the donors 2, 3, and 5.**Enriched ontology terms are illustrated as an enrichment network in which each node represents one specific ontology term. The size of a node is proportional to the total number of genes that fall into this particular ontology term. Enriched terms with a similarity greater than 0.3 are linked by edges, where the thickness of the edges presents the similarity score. The nodes are shown as pie charts, where the size of the differently colored slices depends on the percentage of genes that belong to the three different PDL-specific MSCs subpopulations of donors 2, 3, and 5. Nodes belonging to the same enrichment cluster are grouped and annotated using the best-scored enriched term per cluster as a representative.


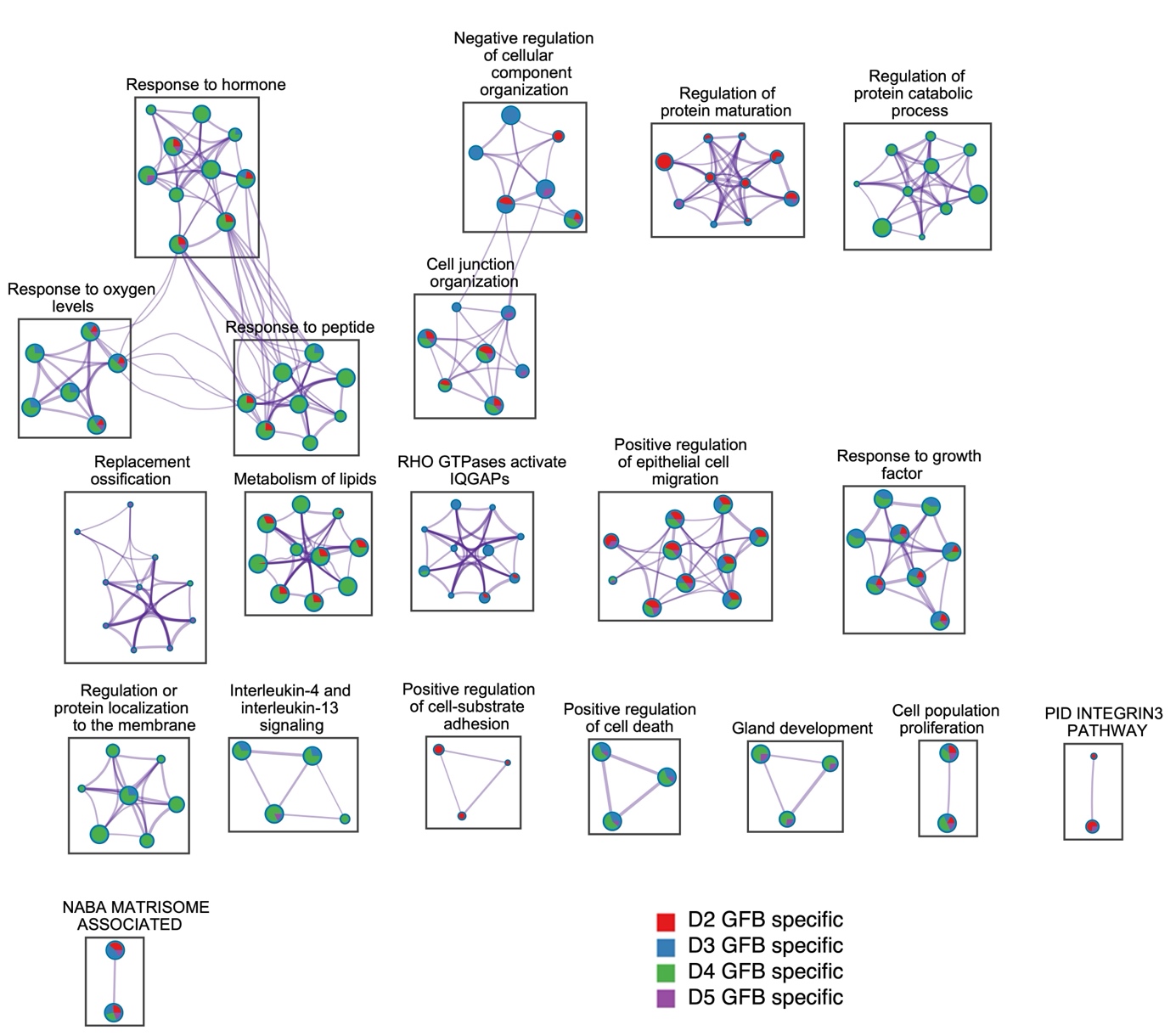
**Appendix Figure 13: Network of enriched ontology terms found in the gingiva-specific MSCs subpopulations of the donors 2, 3, 4, and 5.**Enriched ontology terms are illustrated as an enrichment network in which each node represents one specific ontology term. The size of a node is proportional to the total number of genes that fall into this particular ontology term. Enriched terms with a similarity greater than 0.3 are linked by edges, where the thickness of the edges presents the similarity score. The nodes are shown as pie charts, where the size of the differently colored slices depends on the percentage of genes that belong to the four different gingiva-specific MSCs subpopulations of donors 2, 3, 4, and 5. Nodes belonging to the same enrichment cluster are grouped and annotated using the best-scored enriched term per cluster as a representative.


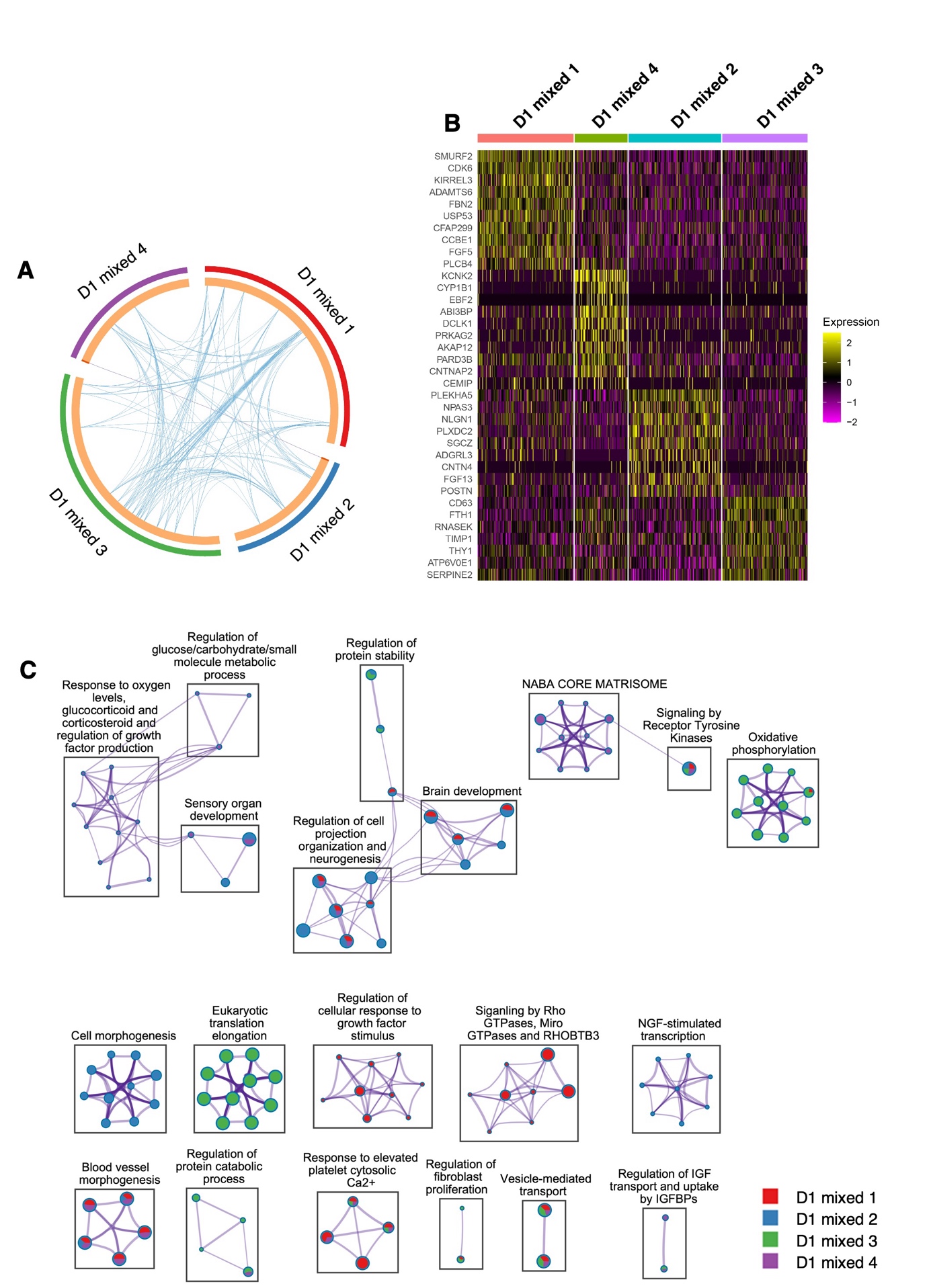
**Appendix Figure 14: DGE and gene/pathway enrichment analysis comparing mixed subpopulations 1-4 found in donor 1.** (A) Circos plot illustrates the functional overlap of significantly upregulated genes between the four mixed subpopulations in donor 1. The light orange arc per subpopulation depicts the uniqueness of the genes in each subpopulation, whereas the blue lines link genes between the subpopulations, which are different but share the same significantly enriched ontology terms. The lists of significantly upregulated genes in the mixed subpopulations 1, 2, 3, and 4 are presented by the red, blue, green, and violet arc, respectively. (B) Gene expression heatmap depicting the top 10 differently expressed genes of individual cells between mixed subpopulations 1-4 in donor 1. The yellow color demonstrates the upregulated, while the pink color represents the downregulated gene expression values. (C) Enriched ontology terms were illustrated as an enrichment network, each node representing one specific ontology term. The size of a node is proportional to the total number of genes that fall into this specific ontology term. Similar terms are linked by edges, where the similarity score is represented by the thickness of the edges. The nodes are presented as pie charts, where the size of the differently colored slices depends on the percentage of genes that belong to the four different mixed MSC subpopulations in donor 1. Nodes belonging to the same enrichment cluster are grouped and annotated using the best-scored enriched term per cluster as a representative. Data information: The mixed subpopulation 5 was excluded from downstream analysis due to its mitotic character.


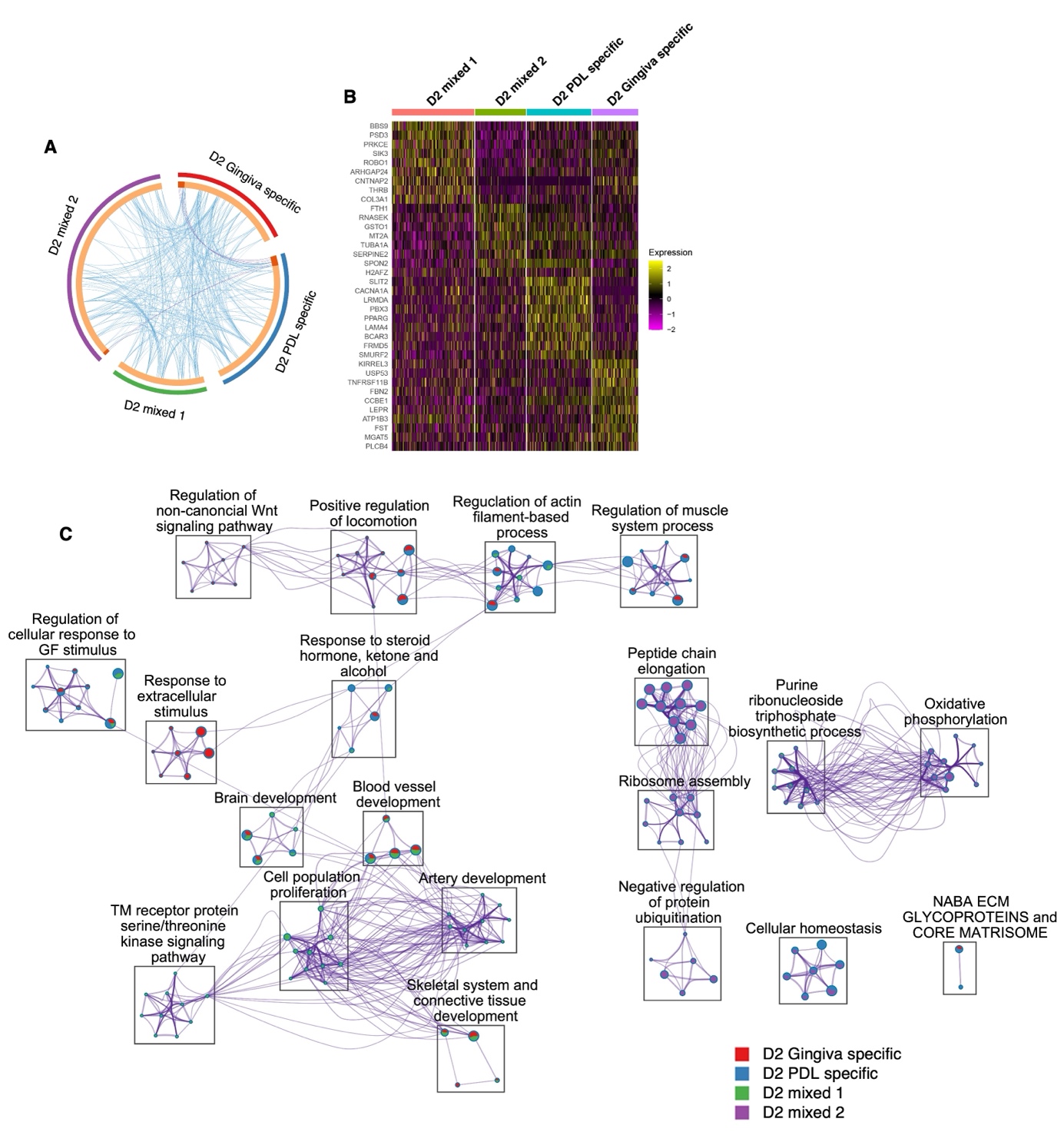


**Appendix Figure 15: DGE and gene/pathway enrichment analysis comparing four subpopulations found in donor 2.** (A) Circos plot illustrates the functional overlap of significantly upregulated genes between four subpopulations in donor 2. The light orange arc per subpopulation depicts the uniqueness of the genes in each subpopulation. Genes that belong to at least two different subpopulations are illustrated in red and are linked by purple lines. The blue lines link genes between different subpopulations that share the same significantly enriched ontology terms. The lists of significantly upregulated genes of the gingiva- and PDL-specific subpopulations and the mixed subpopulations 1, and 2 are presented by the red, blue, green, and violet arc, respectively. (B) Gene expression heatmap depicting the top 10 differently expressed genes of individual cells between four subpopulations in donor 2. The yellow color demonstrates the upregulated, while the pink color represents the downregulated gene expression values. (C) Enriched ontology terms were illustrated as an enrichment network, each node representing one specific ontology term. The size of a node is proportional to the total number of genes that fall into this specific ontology term. Similar terms are linked by edges, where the similarity score is represented by the thickness of the edges. The nodes are presented as pie charts, where the size of the differently colored slices depends on the percentage of genes that belong to the four different MSC subpopulations in donor 2. Nodes belonging to the same enrichment cluster are grouped and annotated using the best-scored enriched term per cluster as a representative. Data information: The mixed subpopulation 3 was excluded from downstream analysis due to their mitotic character.


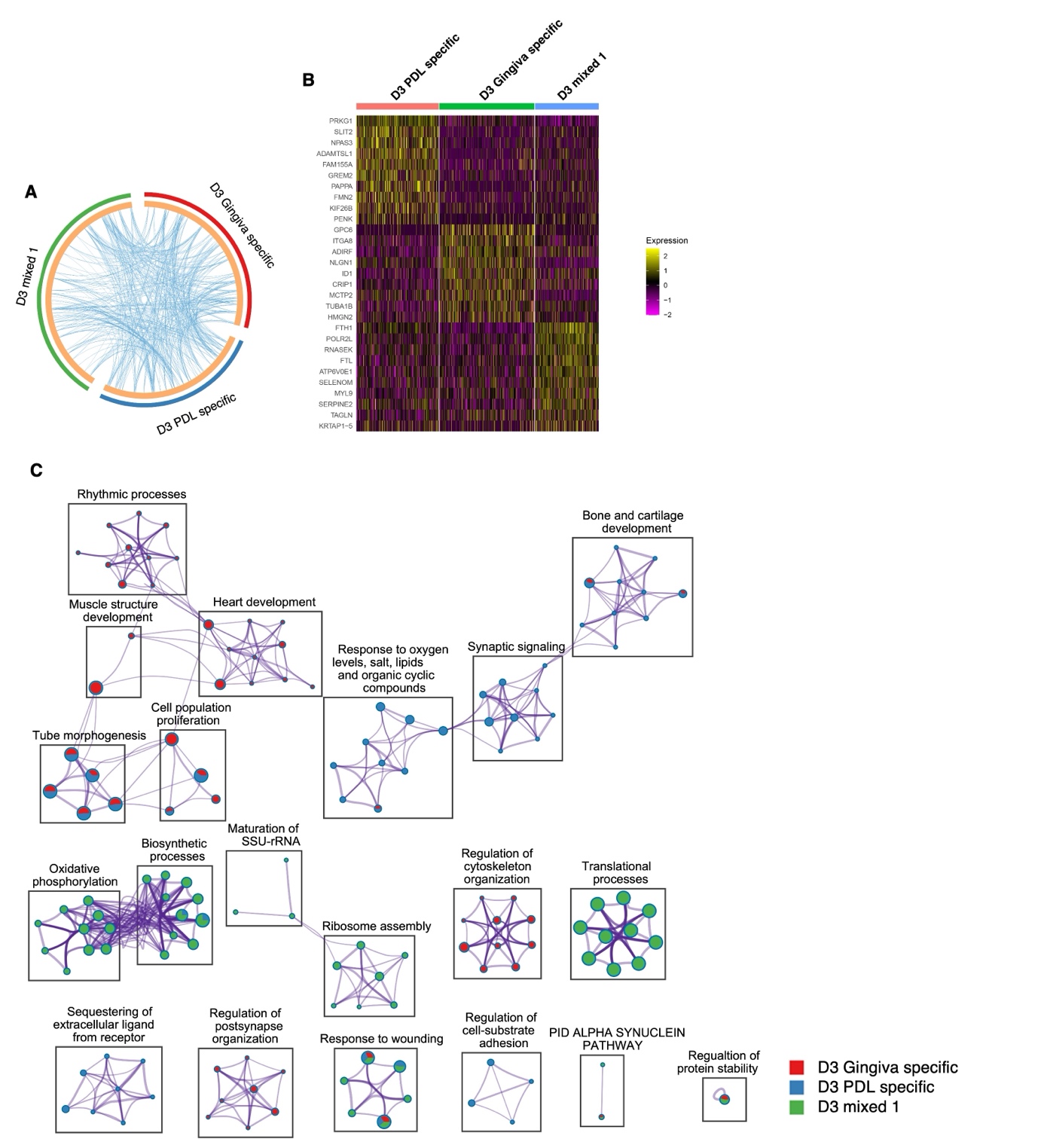
**Appendix Figure 16: DGE and gene/pathway enrichment analysis comparing all subpopulations found in donor 3.** (A) Circos plot illustrates the functional overlap of significantly upregulated genes between all three subpopulations in donor 3. The light orange arc per subpopulation depicts the uniqueness of the genes in each subpopulation. The blue lines link genes between different subpopulations that share the same significantly enriched ontology terms. The lists of significantly upregulated genes of the gingiva- and PDL-specific subpopulations and the mixed subpopulations 1 are presented by the red, blue, and green arc, respectively. (B) Gene expression heatmap depicting the top 10 differently expressed genes of individual cells between all three subpopulations in donor 3. The yellow color demonstrates the upregulated, while the pink color represents the downregulated gene expression values. (C) Enriched ontology terms were illustrated as an enrichment network, each node representing one specific ontology term. The size of a node is proportional to the total number of genes that fall into this specific ontology term. Similar terms are linked by edges, where the similarity score is represented by the thickness of the edges. The nodes are presented as pie charts, where the size of the differently colored slices depends on the percentage of genes that belong to the three different MSC subpopulations in donor 3. Nodes belonging to the same enrichment cluster are grouped and annotated using the best-scored enriched term per cluster as a representative.


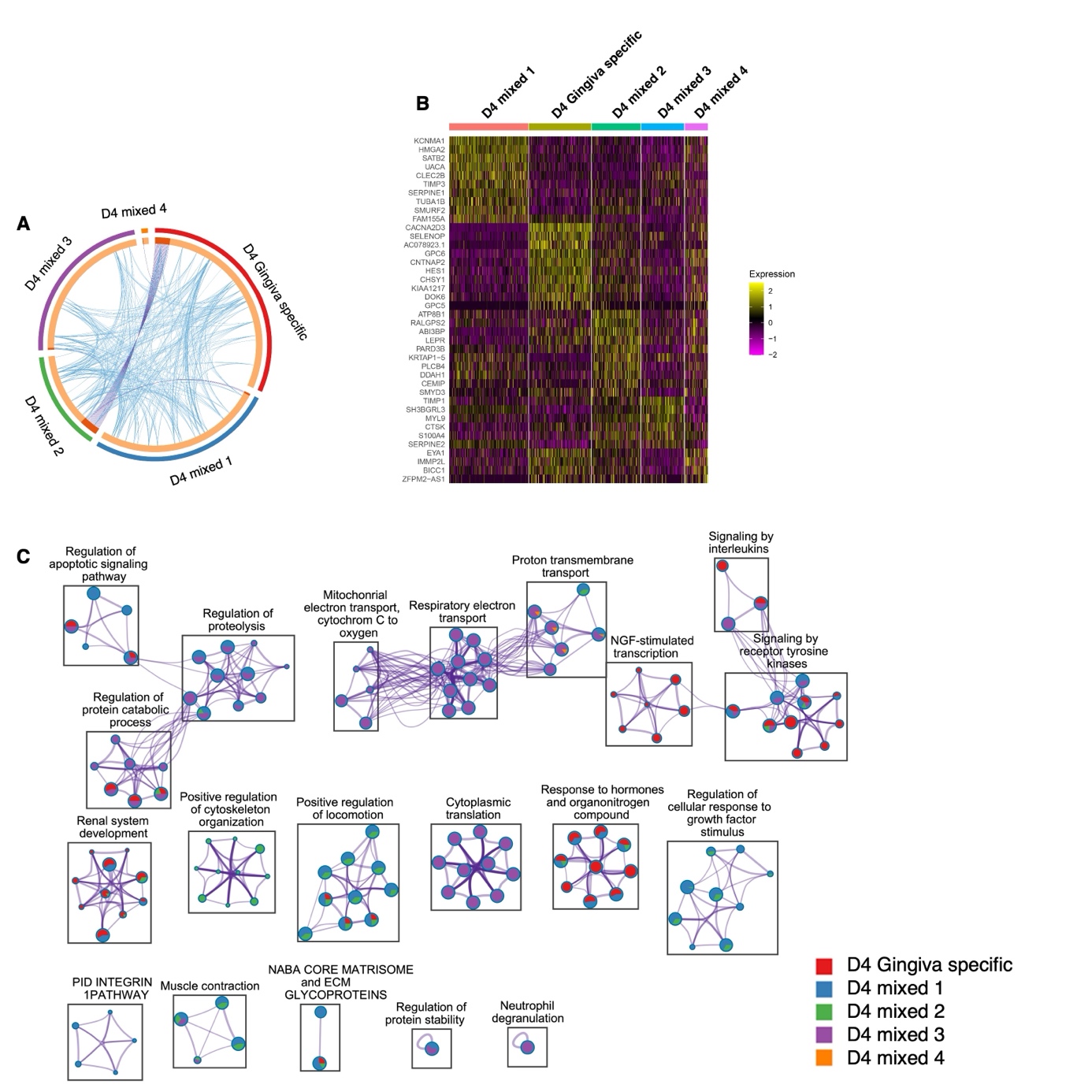


**Appendix Figure 17: DGE and gene/pathway enrichment analysis comparing tissue-specific and mixed subpopulations in donor 4.** (A) Circos plot illustrates the functional overlap of significantly upregulated genes between all five subpopulations in donor 4. The light orange arc per subpopulation depicts the uniqueness of the genes in each subpopulation. Genes that belong to at least two different subpopulations are illustrated in red and are linked by purple lines. The blue lines link genes between different subpopulations that share the same significantly enriched ontology terms. The lists of significantly upregulated genes of the gingiva-specific subpopulation and the mixed subpopulations 1, 2, 3, and 4 are presented by the red, blue, green, violet, and orange arcs, respectively. (B) Gene expression heatmap depicting the top 10 differently expressed genes of individual cells between all five subpopulations in donor 4. The yellow color demonstrates the upregulated, while the pink color represents the downregulated gene expression values. (C) Enriched ontology terms were illustrated as an enrichment network, each node representing one specific ontology term. The size of a node is proportional to the total number of genes that fall into this specific ontology term. Similar terms are linked by edges, where the similarity score is represented by the thickness of the edges. The nodes are presented as pie charts, where the size of the differently colored slices depends on the percentage of genes that belong to the five different MSC subpopulations in donor 4. Nodes belonging to the same enrichment cluster are grouped and annotated using the best-scored enriched term per cluster as a representative.

**
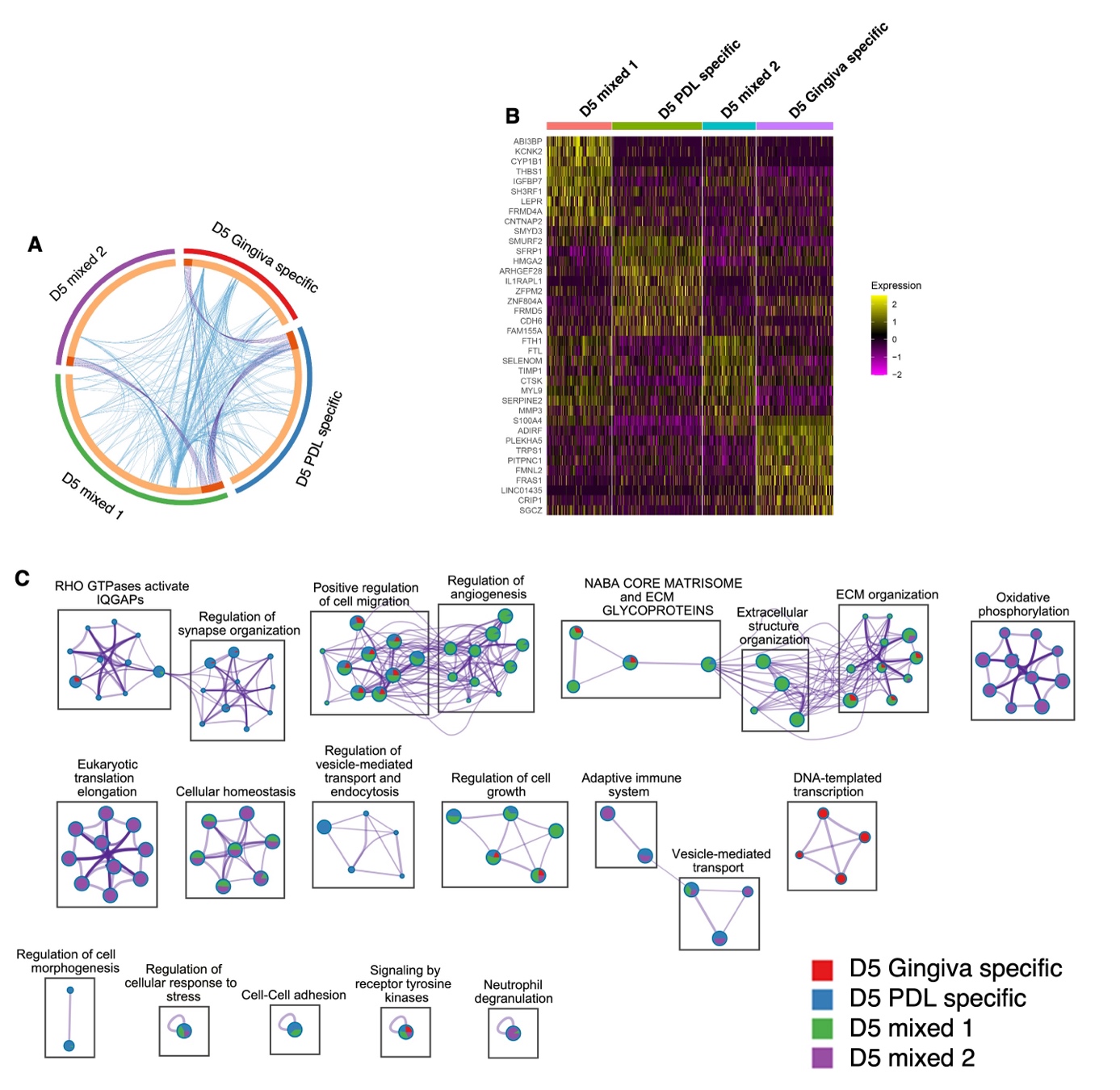
Appendix Figure 18: Differently gene expression and gene/pathway enrichment analysis comparing tissue-specific and mixed subpopulations in donor 5.** (A) Circos plot illustrates the functional overlap of significantly upregulated genes between four subpopulations in donor 5. The light orange arc per subpopulation depicts the uniqueness of the genes in each subpopulation. Genes that belong to at least two different subpopulations are illustrated in red and are linked by purple lines. The blue lines link genes between different subpopulations that share the same significantly enriched ontology terms. The lists of significantly upregulated genes of the gingiva- and PDL-specific subpopulations and the mixed subpopulations 1, and 2 are presented by the red, blue, green, and violet arc, respectively. (B) Gene expression heatmap depicting the top 10 differently expressed genes of individual cells between four subpopulations in donor 5. The yellow color demonstrates the upregulated, while the pink color represents the downregulated gene expression values. (C) Enriched ontology terms were illustrated as an enrichment network, each node representing one specific ontology term. The size of a node is proportional to the total number of genes that fall into this specific ontology term. Similar terms are linked by edges, where the similarity score is represented by the thickness of the edges. The nodes are presented as pie charts, where the size of the differently colored slices depends on the percentage of genes that belong to the four different MSC subpopulations in donor 5. Nodes belonging to the same enrichment cluster are grouped and annotated using the best-scored enriched term per cluster as a representative. Data information: The mixed subpopulation 3 was excluded from downstream analysis due to their mitotic nature.


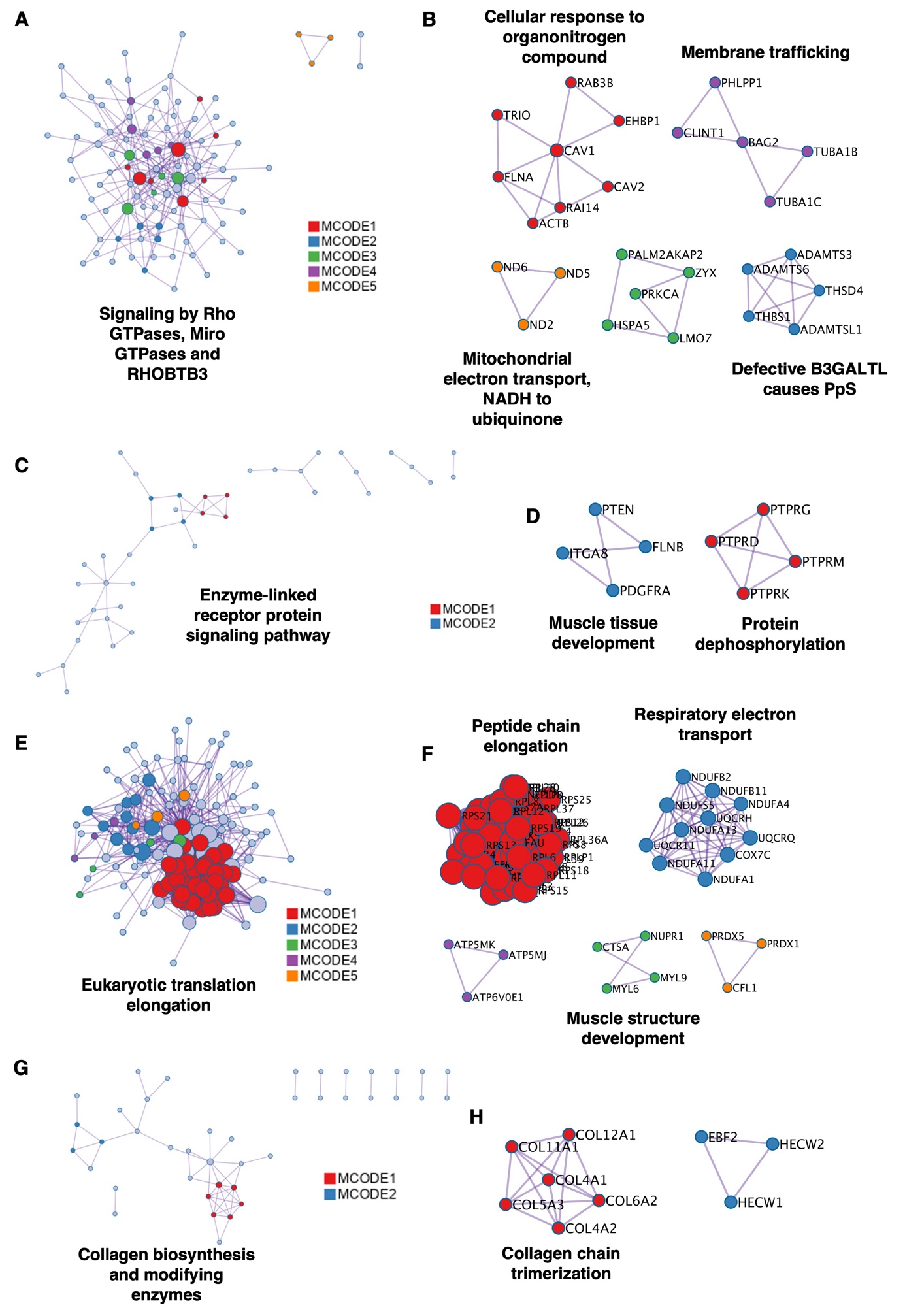
**Appendix Figure 19: Protein-protein interaction (PPI) and MCODE identification within upregulated genes of the mixed subpopulations in donor 1.** (A, C, E, G) Upregulated PPI network identified within the mixed subpopulations 1 (A), 2 (C), 3 (E), and 4 (G). Gene, and pathway enrichment analysis were applied to the identified PPI networks. Based on their log10(P) values, the best-scored biological meaning was chosen. (B, D, F, H) Running the MCODE algorithm on the PPI networks of the mixed subpopulations 1 (B), 2 (D), 3 (F), and 4 (H) identified various numbers of densely connected protein networks (MCODEs). Gene, and pathway enrichment analysis of each MCODE individually deciphered the biological meanings of each MCODE network. Based on their log10(P) values, the best-scored enriched ontology term was chosen. No statistically enriched ontology terms were found for MCODE 3, 4+5, and 2 in the mixed subpopulations 1, 3, and 4, respectively.


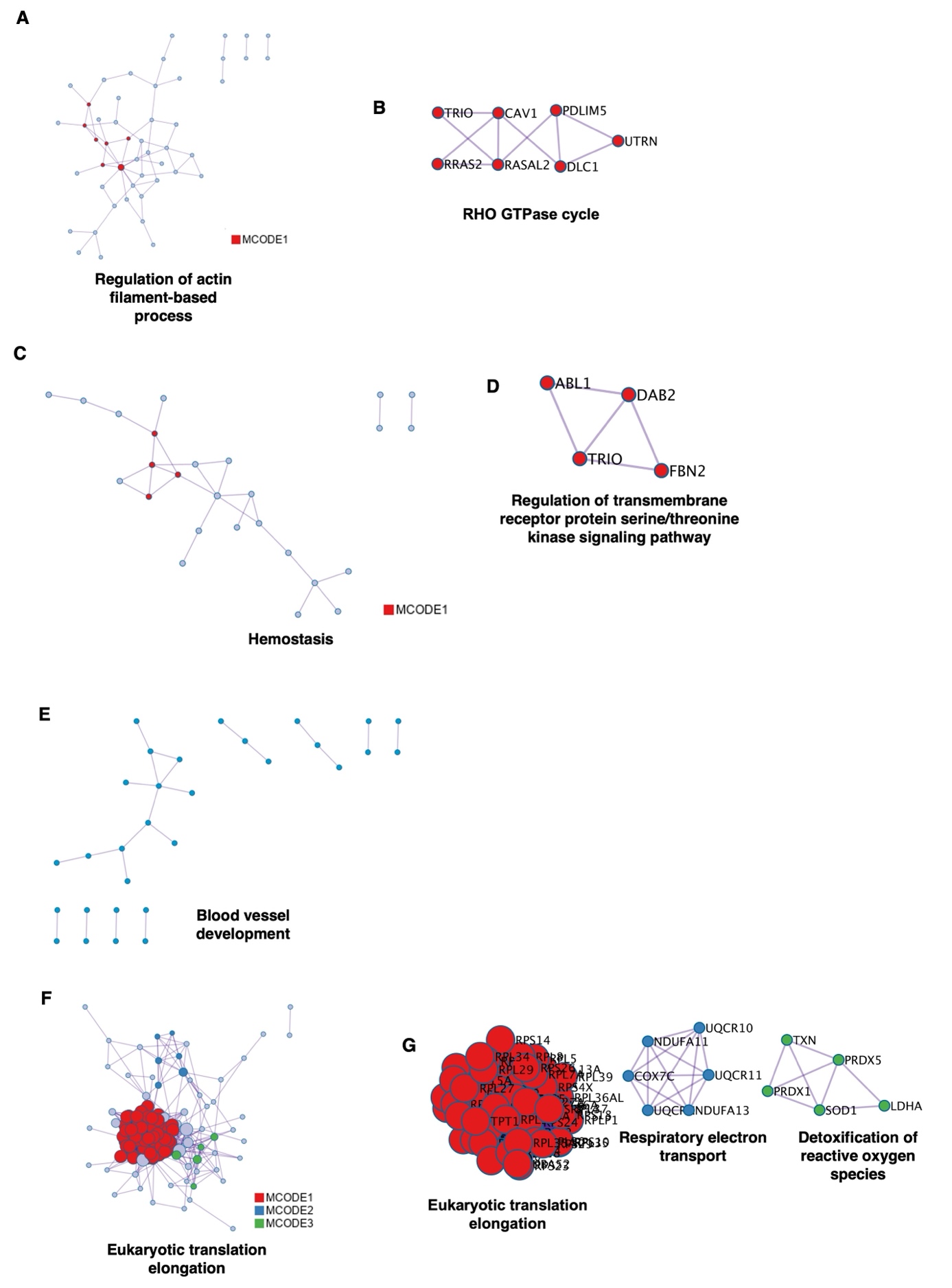
**Appendix Figure 20: PPI and MCODE identification within upregulated genes of the tissue-specific and mixed subpopulations in donor 2.** (A, C, E, F) Upregulated PPI networks identified within the PDL- (A), and gingiva-(C) specific subpopulations and the mixed subpopulations 1 (E), and 2 (F). Gene, and pathway enrichment analysis was applied to the identified PPI networks. Based on their log10(P) values, the best-scored biological meaning was chosen. (B, D, G) Running the MCODE algorithm on the PPI networks of the PDL- (B), and gingiva- (D) specific subpopulations and the mixed subpopulation 1, and 2 (G) identified various numbers of densely connected protein networks (MCODE). For the PPI network in the mixed subpopulation 1 no MCODE was detected. Gene and pathway enrichment analysis of each MCODE individually deciphered the biological meanings of each MCODE network. The best-scored enriched ontology term was chosen based on their log10(P) values.


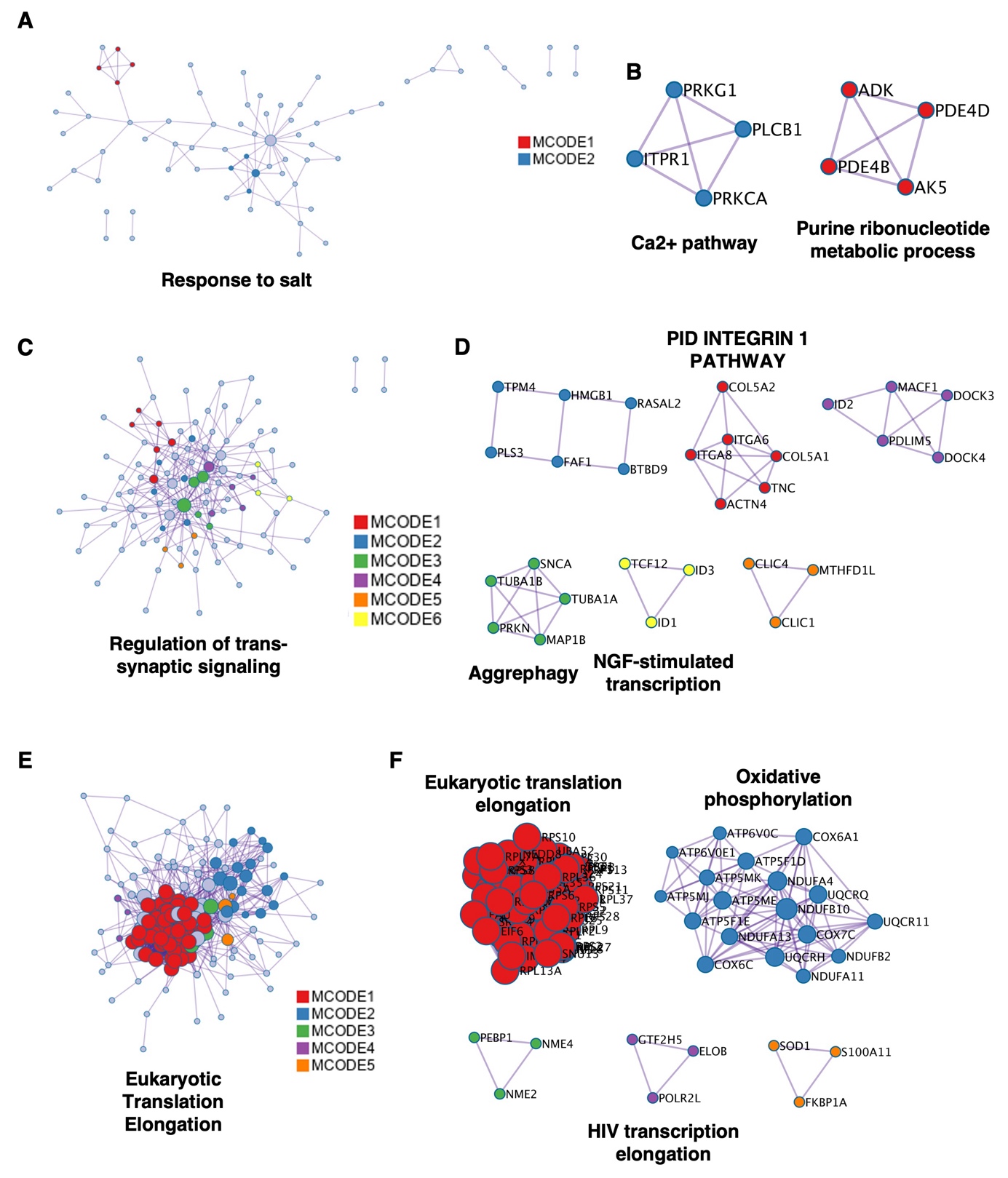
**Appendix Figure 21: PPI and MCODE identification within upregulated genes of the tissue-specific and the mixed subpopulation in donor 3.** (A, C, E) Upregulated PPI networks identified within the PDL- (A), and gingiva- (C) specific subpopulations and the mixed subpopulation (E). Gene, and pathway enrichment analysis was applied to the identified PPI networks. Based on their log10(P) values, the best-scored biological meaning was chosen. (B, D, F) Running the MCODE algorithm on the PPI networks of the PDL- (B), and gingiva- (D) specific subpopulations and on the mixed subpopulation 1 (F) identified various numbers of densely connected protein networks (MCODE). Gene and pathway enrichment analysis of each MCODE individually deciphered the biological meanings of each MCODE network. Based on their log10(P) values, the best-scored enriched ontology term was chosen. No statistically enriched ontology terms were found for MCODE 2+4+5, and MCODE 3+5 in the GFB-specific subpopulation and the mixed subpopulation 1, respectively.


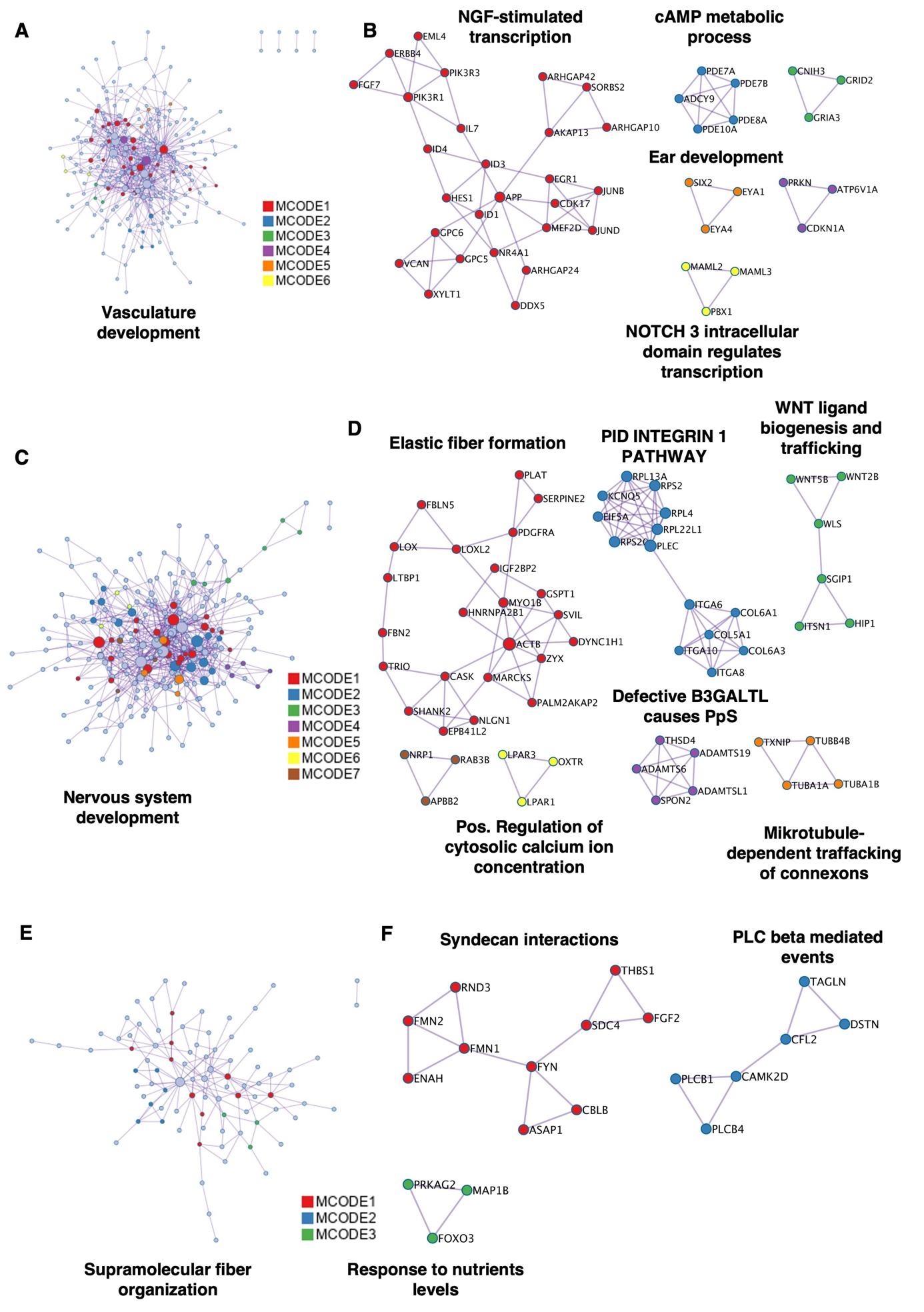


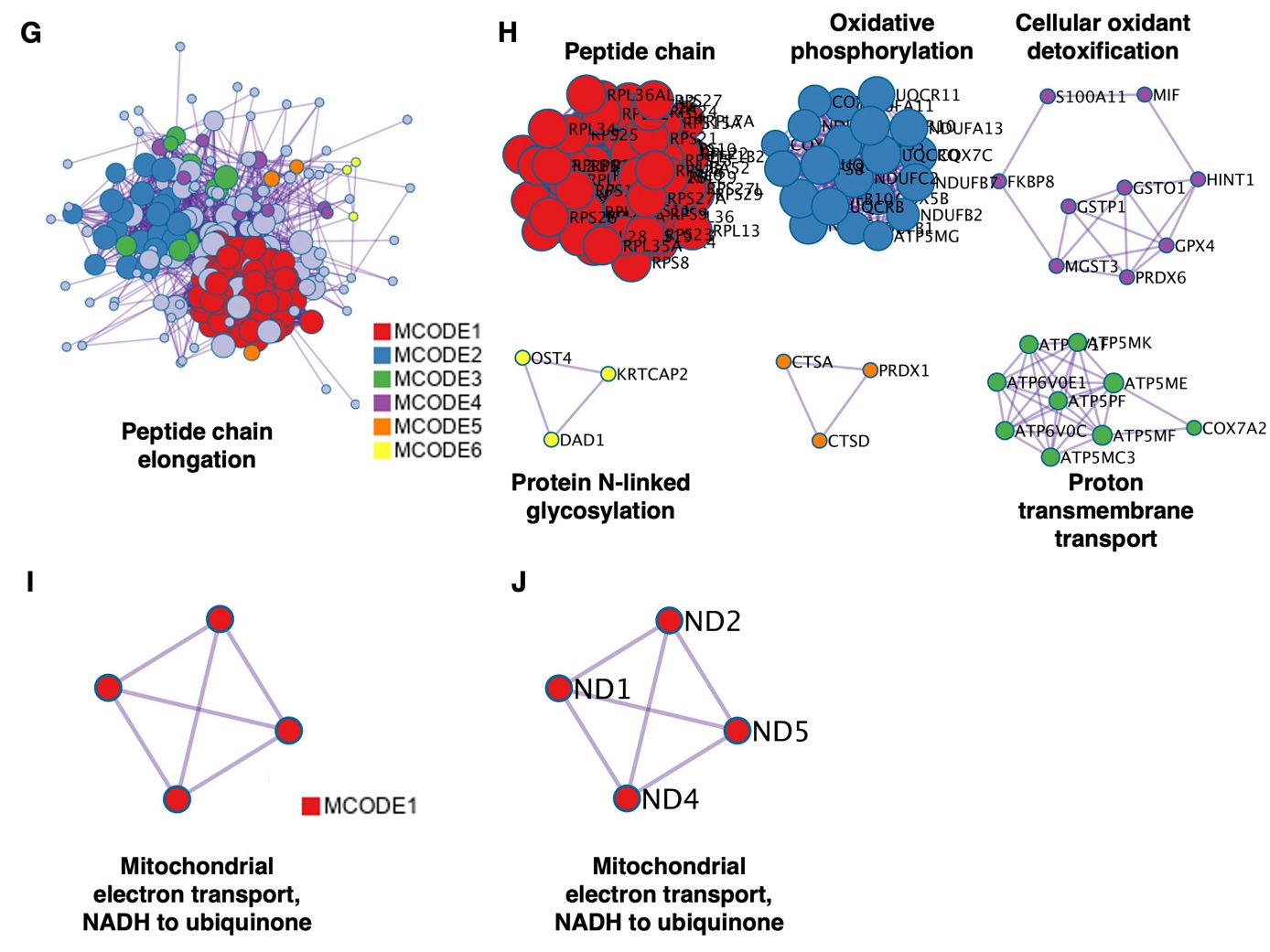
**Appendix Figure 22: PPI and MCODE identification within upregulated genes of the tissue-specific and the mixed subpopulations in donor 4.** (A, C, E, G, I) Upregulated PPI networks identified within the gingiva- (A) specific subpopulation and the mixed subpopulations 1 (C), 2 (E), 3 (G), and 4 (I). Gene, and pathway enrichment analysis was applied to the identified PPI networks. Based on their log10(P) values, the best-scored biological meaning was chosen. (B, D, F, H, J) Running the MCODE algorithm on the PPI networks of the gingiva- (B) specific subpopulation and the mixed subpopulations 1 (D), 2 (F), 3 (H), and 4 (J) identified various numbers of densely connected protein networks (MCODE). Gene and pathway enrichment analysis of each MCODE individually deciphered the biological meanings of each MCODE network. Based on their log10(P) values, the best-scored enriched ontology term was chosen. No statistically enriched ontology terms were found for MCODE 3+4, MCODE 7, and MCODE 5 in the gingiva-specific subpopulation and the mixed subpopulation 1, and 3, respectively.


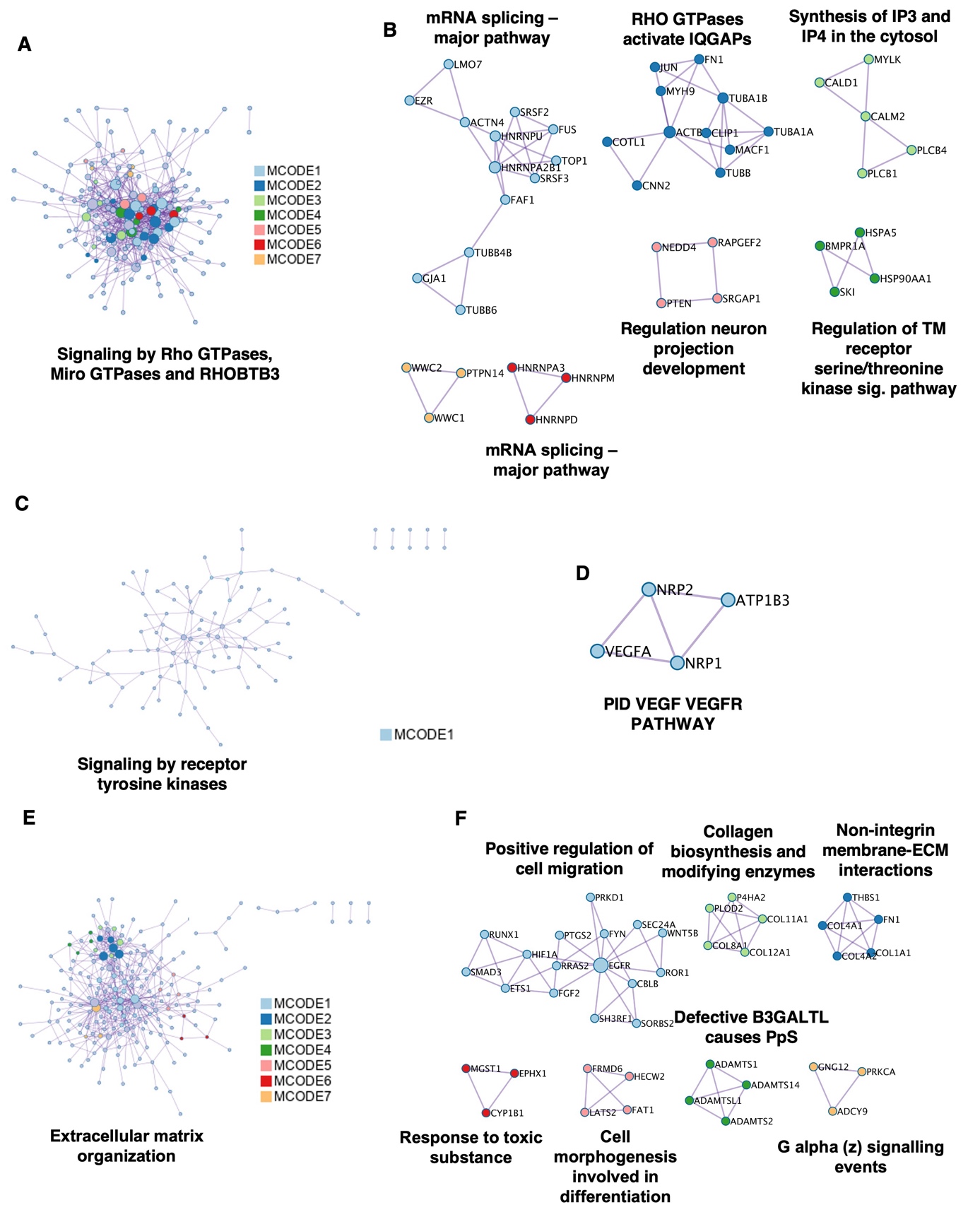


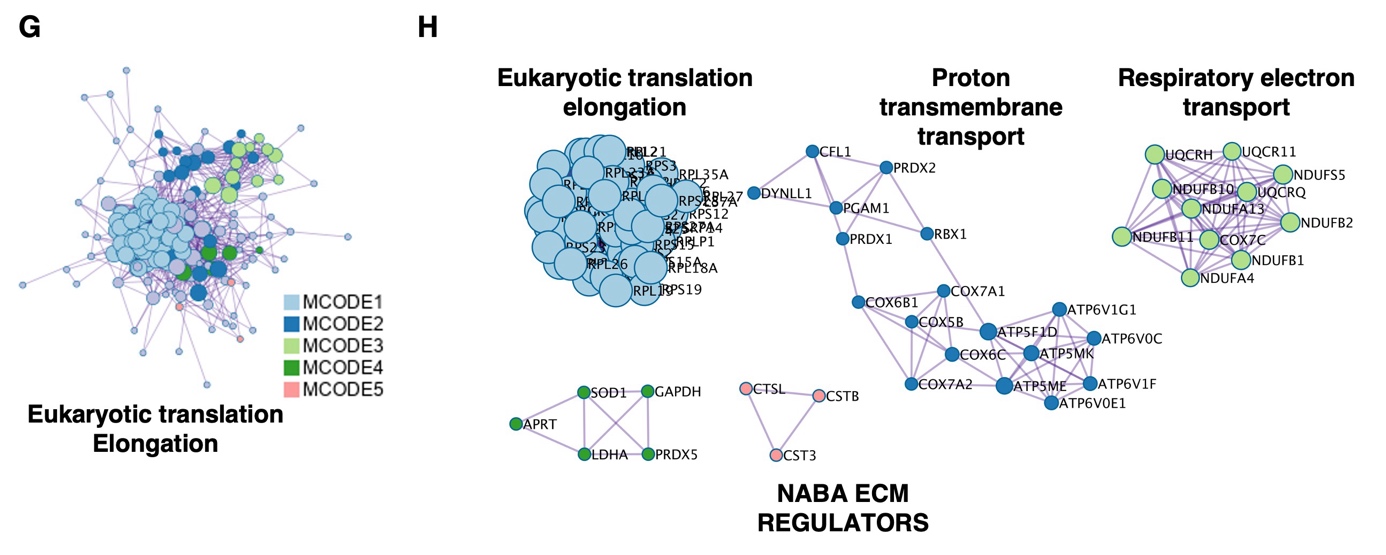
**Appendix Figure 23: PPI and MCODE identification within upregulated genes of the tissue-specific and the mixed subpopulations in donor 5.** (A, C, E, G) Upregulated PPI networks identified within the PDL- (A), and gingiva- (C) specific subpopulations and the mixed subpopulations 1 (E), and 2 (G). Gene and pathway enrichment analysis was applied to the identified PPI networks. The best-scored biological meaning was chosen based on their log10(P) values. (B, D, F, H) Running the MCODE algorithm on the PPI networks of the PDL- (B) and gingiva- (D) specific subpopulations and the mixed subpopulations 1 (F) and 2 (H) identified various numbers of densely connected protein networks (MCODE). Gene and pathway enrichment analysis of each MCODE individually deciphered the biological meanings of each MCODE network. The best-scored enriched ontology term was chosen based on their log10(P) values. No statistically enriched ontology terms were found for MOCDE 7 and MCODE 4 in the PDL-specific and mixed subpopulations 2, respectively.

| **Hashtag Antibody** | **Sample** | **Recognized antigens** | **Clones** | **Barcode sequence** | |
| --- | --- | --- | --- | --- | --- |
| Total Seq-A 0255 | Donor 1 – PDL | CD298 β2 microglobulin | LNH-94 2M2 | AAGTATCGTTTCGCA |  |
| Total Seq-A 0254 | Donor 2 – PDL | CD298 β2 microglobulin | LNH-94 2M2 | AGTAAGTTCAGCGTA |  |
| Total Seq-A 0253 | Donor 3 – PDL | CD298 β2 microglobulin | LNH-94 2M2 | TTCCGCCTCTCTTTG |  |
| Total Seq-A 0252 | Donor 4 – PDL | CD298 β2 microglobulin | LNH-94 2M2 | TGATGGCCTATTGGG |  |
| Total Seq-A 0251 | Donor 5 – DL | CD298 β2 microglobulin | LNH-94 2M2 | GTCAACTCTTTAGCG |  |
| Total Seq-A 0260 | Donor 1 – Gingiva | CD298 β2 microglobulin | LNH-94 2M2 | ATTGACCCGCGTTAG |  |
| Total Seq-A 0257 | Donor 2 – Gingiva | CD298 β2 microglobulin | LNH-94 2M2 | TGTCTTTCCTGCCAG |  |
| Total Seq-A 0259 | Donor 3 – Gingiva | CD298 β2 microglobulin | LNH-94 2M2 | CAGTAGTCACGGTCA |  |
| Total Seq-A 0256 | Donor 4 – Gingiva | CD298 β2 microglobulin | LNH-94 2M2 | GGTTGCCAGATGTCA |  |
| Total Seq-A 0258 | Donor 5 – Gingiva | CD298 β2 microglobulin | LNH-94 2M2 | CTCCTCTGCAATTAC |  |

**Appendix Table 1.** Information about the recognized antigens, clones, and the barcode sequence of the used Total Seq-A Hashtag antibodies and about which Hashtag antibody was used to label which sample.

| **Donor source** | **Tissue source** | **Sex** | **Age** | **Used passage** | **Oral health of the donor** |
| --- | --- | --- | --- | --- | --- |
| Donor 1 | PDL/Gingiva | Female | 22 | P5 | Periodontally healthy |
| Donor 2 | PDL/Gingiva | Female | 20 | P6 | Periodontally healthy |
| Donor 3 | PDL/Gingiva | Male | 20 | P5 | Periodontally healthy |
| Donor 4 | PDL/Gingiva | Female | 18 | P6 | Periodontally healthy |
| Donor 5 | PDL/Gingiva | Male | 17 | P4 | Periodontally healthy |

**Appendix Table 2.** Donor and tissue source-specific information of all 10 samples. PDL, periodontal ligament; P, passage;

|  | **PDL_D1** | **PDL_D2** | **PDL_D3** | **PDL_D4** | **PDL_D5** | **Gingiva_D1** | **Gingiva_D2** | **Gingiva_D3** | **Gingiva_D4** | **Gingiva_D5** |
| --- | --- | --- | --- | --- | --- | --- | --- | --- | --- | --- |
| **Cells** |  |  |  |  |  |  |  |  |  |  |
| Cell number | 327 | 290 | 383 | 335 | 457 | 290 | 421 | 402 | 470 | 469 |
| Median reads per cell | 17,424 | 17,307 | 15,426 | 21,708 | 16,723 | 16,750 | 16,235 | 14,706 | 19,011 | 16,895 |
| Median genes per cell | 3,777 | 3,653 | 3,425 | 4,254 | 3,756 | 3,628 | 3,554 | 3,373 | 3,899 | 3,633 |
| Total genes detected | 20,374 | 19,790 | 20,564 | 21,164 | 21,340 | 20,610 | 20,883 | 20,631 | 22,264 | 21,586 |
| Median UMI counts per cell | 11,240 | 11,185 | 9,793 | 14,169 | 10,812 | 10,716 | 10,487 | 9,464 | 12,085 | 11,076 |
|  |  |  |  |  |  |  |  |  |  |  |
| **Cell multiplexing** |  |  |  |  |  |  |  |  |  |  |
| Cells assigned to this sample | 6.86% | 6.08% | 8.04% | 7.03% | 9.59% | 6.08% | 8.83% | 8.43% | 9.86% | 9.84% |
| Cells assigned to other samples | 73.79% | 74.57% | 72.62% | 73.63% | 71.07% | 74.57% | 71.82% | 72.22% | 70.79% | 70.81% |
| Cell-associated barcodes not assigned any CMOs | 1.68% | 1.68% | 1.68% | 1.68% | 1.68% | 1.68% | 1.68% | 1.68% | 1.68% | 1.68% |
| Cell-associated barcodes identified as multiplets | 17.67% | 17.67% | 17.67% | 17.67% | 17.67% | 17.67% | 17.67% | 17.67% | 17.67% | 17.67% |
|  |  |  |  |  |  |  |  |  |  |  |
| **Mapping Metrics** |  |  |  |  |  |  |  |  |  |  |
| Number of reads from cells called from this sample | 6,027,337 | 5,177,053 | 6,420,757 | 7,488,804 | 8,208,728 | 5,167,921 | 7,130,525 | 6,373,794 | 9,320,385 | 8,704,945 |
| Mapped to genome | 96.78% | 96.75% | 96.77% | 96.80% | 96.69% | 96.76% | 96.69% | 96.71% | 96.73% | 96.77% |
| Confidently mappged to genome | 93.90% | 93.92% | 93.77% | 93.89% | 93.70% | 93.72% | 93.77% | 93.76% | 93.62% | 93.87% |
| Confidently mapped to transcriptome | 81.53% | 81.23% | 80.95% | 82.39% | 81.02% | 81.34% | 80.46% | 80.38% | 81.12% | 81.56% |
| Confidently mapped to intronic regions | 19.18% | 18.93% | 19.08% | 18.00% | 19.66% | 19.13% | 19.88% | 19.44% | 19.30% | 18.57% |
| Confidently mapped to exonic regions | 70.44% | 70.38% | 70.11% | 71.63% | 69.47% | 70.08% | 68.96% | 69.59% | 69.54% | 70.63% |
| Confidently mapped to intergenic regions | 4.28% | 4.61% | 4.58% | 4.27% | 4.57% | 4.51% | 4.93% | 4.73% | 4.78% | 4.67% |
| Confidently mapped antisense | 7.53% | 7.55% | 7.70% | 6.68% | 7.55% | 7.31% | 7.83% | 8.10% | 7.12% | 7.09% |

**Appendix Table 3.** Sequencing output information per donor. PDL, periodontal ligament; D, Donor; CMO, cell multiplexing oligo.

|  | **Cell statistics** |
| --- | --- |
| Estimated number of cells | 4,766 |
| Mean reads per cell | 22,588 |
| Cells assigned to a sample | 3,844 |
| Cell-associated barcodes not assigned to any CMOs | 80 |
| Cell-associated barcodes identified as multiplets | 842 |
|  |  |
|  | **Sequencing Metrics** |
| Number of reads | 107,653,624 |
| Number of short reads skipped | 0 |
| Q 30 barcodes | 93.10% |
| Q 30 UMI | 92.60% |
| Q 30 RNA read | 91.20% |
|  |  |
|  | **Mapping Metrics (amongst all reads in library)** |
| Number of reads in the library | 107,653,624 |
| Mapped to genome | 96.13% |
| Confidently mappged to genome | 92.86% |
| Confidently mapped to transcriptome | 80.54% |
| Confidently mapped to intronic regions | 18.72% |
| Confidently mapped to exonic regions | 69.70% |
| Confidently mapped to intergenic regions | 4.44% |
| Confidently mapped antisense | 7.33% |
|  |  |
|  | **Metrics per physical library** |
| Number of reads | 107,653,624 |
| Valid barcodes | 97.41% |
| Vaild UMIs | 99.91% |
| Sequencing saturation | 20.22% |
| Confidently mapped reads in cells | 95.98% |
| Mean reads per cell | 22,588 |

**Appendix Table 4.** Single-cell RNA-seq information. Library
 information – gene expression. CMO, cell multiplexing oligo;
 UMI, unique molecular identifier;

|  | **Multiplexing metrics** |
| --- | --- |
| Estimated number of cell-associated barcodes | 4,766 |
| Number of samples assigned at least one cell | 10 |
| Cells assigned to a sample | 3,844 |
| Singlet capture ratio | 0.84 |
| Cell-associated barcodes identified as multiplets | 842 |
| Median CMO UMIs per cell | 278 |
|  |  |
|  | **Multiplexing sample assignments** |
| Mean reads per cell-associated barcode | 2,130 |
| Median CMO UMIs per cell-associated barcode | 319 |
|  |  |
|  | **Sequencing Metrics** |
| Number of reads | 12,005,340 |
| Number of short reads skipped | 0 |
| Q 30 barcodes | 93.30% |
| Q 30 UMI | 92.70% |
| Q 30 RNA read | 61.80% |
|  |  |
|  | **Metrics per physical library** |
| Number of reads | 12,005,340 |
| Valid barcodes | 99.01% |
| Valid UMIs | 99.92% |
| Sequencing saturation | 75.59% |
| Fraction reads in cell-associated barcodes | 58.04% |
| Mean reads per cell-associated barcode | 2,130 |
| Fraction CMO reads | 85.26% |
| Fraction CMO reads usable | 49.09% |
| Fraction unrecognized CMO | 14.74% |
| Fraction reads from multiplets | 0.00% |

**Appendix Table 5.** Single-cell RNA-seq information. Library
information-multiplexing. CMO, cell multiplexing oligo;
UMI, unique molecular identifier;

| **CMO Name** | **Fraction reads in cell-associated barcodes** | **Cells assigned to CMO** | **CMO signal-to-noise ratio** |
| --- | --- | --- | --- |
| PDL_D1 | 72.44% | 8.51% | 8.12% |
| PDL_D2 | 79.35% | 7.54% | 8.69% |
| PDL_D3 | 42.45% | 9.96% | 7.03% |
| PDL_D4 | 55.58% | 8.71% | 7.38% |
| PDL_D5 | 48.60% | 11.89% | 7.10% |
| Gingiva_D1 | 39.25% | 7.54% | 6.67% |
| Gingiva_D2 | 70.44% | 10.95% | 8.58% |
| Gingiva_D3 | 65.58% | 10.46% | 8.31% |
| Gingiva_D4 | 58.57% | 12.23% | 6.97% |
| Gingiva_D5 | 51.67% | 12.20% | 6.62% |

**Appendix Table 6.** Single-cell RNA-seq information. Sequencing
metrics per CMO. PDL, periodontal ligament; D, donor;
CMO, cell multiplexing oligo;

**Material and Methods**

**MSCs isolation and cultivation**

Primary human MSCs were isolated from the PDL and gingiva obtained from five extracted third molars. The teeth were extracted from five periodontally healthy individuals (two males and three females, aged between 17 and 22 at the time of extraction, Appendix Table 2), obtaining five PDL- and gingiva-derived MSCs samples from the same donors (in total n=10). The five individuals were periodontally healthy, showing no sign of periodontitis or gingival inflammation, had no periodontal treatment within the last three months, had no systemic disease, acute infection, immune suppressive or immunomodulatory or anti-inflammatory medication and antibiotics intake, had no immunodeficiency and no history of radio- and chemotherapy.

Before the surgical procedure, all patients, or their parents if applicable, signed an informed consent form. The Ethics Committee of the Medical University of Vienna approved the MSCs’ isolation and all experimental techniques within this study. They were conducted in compliance with the Declaration of Helsinki and the Good Scientific Practice Guidelines of the Medical University of Vienna.
After extraction, the teeth were placed in Dulbecco’s modified Eagle’s medium (DMEM, Capricorn Scientific, Ebsdorfergrund, Germany) supplemented with 10% fetal bovine serum (FBS), 100μg/ml streptomycin, and 100U/ml penicillin (Capricorn Scientific) and immediately transferred to the laboratory. After cleaning the teeth with 1x phosphate-buffered saline (1xPBS), the gingiva was cut into small pieces, and the PDL was scrapped off from the mid-third of the tooth’s root. Minced PDL and gingiva tissue were cultured at 37° Celsius, 5% CO_2_, and 95% humidity using DMEM supplemented as described above. After the cell’s outgrowth from the tissue pieces, MSCs were harvested, expanded, and cultured under the conditions described above. Depending on the donor and tissue sources, passages 4 to 6 were used for the scRNA-seq analysis. Donor and tissue source-specific information is shown in Appendix Table 2.

**MSCs surface antigen analysis**

According to the minimal criteria for MSCs, defined by the International Society for Cell and Gene Therapy (ISCT), MSCs must express specific surface antigens (e.g., CD29, CD73, CD90, CD104, and CD146) and lack the expression of hematopoietic markers (e.g., CD31, CD34, and CD45) (Viswanathan et al. 2019). Hence, cultured PDL- and gingiva-derived MSCs were harvested, and 250.000 cells were used for surface antigen analysis. After washing the cells with 3% bovine serum albumin (BSA) buffer (supplemented with 0.09% sodium azide in 1xPBS), the single-cell suspensions were stained for 30 minutes at room temperature. The following antibodies were used in a 1:10 dilution (all from ThermoFisher Scientific, Waltham, MA, USA): phycoerythrin- (PE-) conjugated mouse anti-human CD29, PE-conjugated mouse anti-human CD73, PE-conjugated mouse anti-human CD90, PE-conjugated mouse anti-human CD105, PE-conjugated mouse anti-human CD146, fluorescein isothiocyanate- (FITC-) conjugated mouse anti-human CD31, FITC-conjugated mouse anti-human CD34 and FITC-conjugated mouse anti-human CD45. Appropriate unlabelled MSCs served as the negative controls. Fluorescence labeling was analyzed by the Attune NxT flow cytometer (ThermoFisher Scientific), in which PE and FITC fluorophores were excited at 488nm. In total, 20,000 cells were acquired per sample. After excluding coincidence events and cell debris, the percentage of positive MSCs was determined using the FCS Express 7 Research software (v7.18.0015., De Novo Software, Pasadena, CA, USA). The data are presented as individual histograms per surface marker and donor and were generated by FCS Express 7 Research software. The corresponding violin plots were created by GraphPad Prism (v9.5.1.).

**Osteogenic, chondrogenic, and adipogenic differentiation analysis**

According to the minimal criteria by ISCT (Viswanathan et al. 2019), MSCs must differentiate into osteoblasts, chondrocytes, and adipocytes *in vitro*. Hence, osteogenic, chondrogenic, and adipogenic differentiation potentials were verified by Alizarin Red, Alcian Blue, and Oil Red staining, respectively. For osteogenic and adipogenic differentiation, 2x10^4^ MSCs were seeded per well in a 24-well format using 500μl DMEM, supplemented with 10% FBS, 100μg/ml streptomycin, and 100U/ml penicillin. After four days of incubation, osteogenic and adipogenic differentiation was started by replacing the DMEM medium with osteogenic (α-MEM supplemented with 1% L-glutamine, 100μg/ml streptomycin, 100U/ml penicillin, 10% FBS, 0.1μM dexamethasone, 50μM ascorbic acid, and 10mM β-glycerophosphate) or complete StemPro^®^ (ThermoFisher Scientific) Adipogenesis differentiation media (supplemented with 100g/ml streptomycin and 100U/ml penicillin), respectively. MSCs further cultured in DMEM medium (with 10% FBS, 100μg/ml streptomycin, and 100U/ml penicillin) served as appropriate negative controls. 250μl of the medium per well was replaced with appropriate fresh medium every three to four days. After 21 and 28 days of osteogenic and adipogenic differentiation, Alizarin Red and Oil Red staining were performed, respectively.

For chondrogenic differentiation, micromasses were formed by seeding 1.6x10^7^ MSCs/ml in 5µl droplets in U-bottom-shaped 96-well plates. After 2 hours of incubation, 200µl complete StemPro^®^ (ThermoFisher Scientific) Chondrogenesis differentiation media (supplemented with 100μg/ml streptomycin and 100U/ml penicillin) were added. MSCs cultured in DMEM medium (with 10% FBS, 100μg/ml streptomycin, and 100U/ml penicillin) served as appropriate controls. The medium was changed every two to three days. After 21 days of chondrogenic differentiation, Alcian Blue staining was performed.

**Alizarin Red, Oil Red O, and Alcian Blue stainings**

To label calcium nodules after osteogenic differentiation, MSCs were washed with 1xPBS. Cells were fixed with cold 70% ethanol, and after washing the wells three times with distilled water, calcium deposits were stained with 1% Alizarin Red staining solution (Thermo Fisher Scientific). Fixation and Alizarin Red staining solutions were incubated for 40 minutes at room temperature, followed by washing the wells four times with distilled water.

To label neutral lipids after adipogenic differentiation, MSCs were washed with 1xPBS, followed by the addition of 4% paraformaldehyde (Thermo Fisher Scientific). After 30 minutes of incubation at room temperature, cells were washed with water and incubated with 60% isopropanol for 5 minutes. Cells were stained with Oil Red (Thermo Fisher Scientific) staining solution (3mg/ml Oil red stock solution diluted 3:2 with water) for 20 minutes at room temperature. MSCs were washed twice with water. To label proteoglycans after chondrogenic differentiation, MSCs were washed with 1xPBS and fixed with 4% paraformaldehyde (Thermo Fisher Scientific) for 30 minutes at room temperature. After washing the cells with 1xPBS, proteoglycans were stained with 1% Alcian Blue (Thermo Fisher Scientific) staining solution (in 0.1 N hydrochloric acid) for 30 minutes at room temperature. Micromasses were washed three times with 0.1 N hydrochloric acid. Alizarin Red and Oil Red stainings were analyzed using an ECHO Revolve microscope (Echo, San Diego, CA, USA) with the 4-fold and 20-fold magnification objectives, respectively. The phase contrast mode was used for oil red staining. For Alcian Blue staining, brightfield pictures were taken using a Leica DMi8 microscope (Leica Microsystems, Wetzlar, Germany) using a 20-fold magnification objective.

For the semi-quantification of the Alizarin Red stain, 10% cetylpyridinium chloride (CPC, Sigma-Aldrich, St. Louis, MO, USA) was added to the wells for 40 minutes at room temperature. The Alcian Blue dye was eluted by incubating micromasses in 6M guanidine-hydrochloric acid overnight at 4°C. The absorbances at 550nm and 600nm were measured for the Alizarin Red and Alcian Blue stains using the Synergy HTX multi-plate reader (BioTek Instruments, Winooski, VT, USA), respectively. 10% CPC and 6M guanidine-hydrochloric acid (Merck Millipore, Burlington, VT, USA) served as blanks. Oil Red staining was not quantified. Statistically significant differences between control and osteogenic/chondrogenic conditions were determined by the two-tailed paired t-test for normally distributed data. Nonparametric data were analyzed using the two-tailed Wilcoxon Signed Ranks test for pairwise comparison. P-values higher than 0.05 were statistically significant. The Kolmogorov-Smirnov test was used to verify the normal distribution.

**MSCs preparation for scRNA-seq analysis**

After harvesting *in vitro* cultured PDL- and gingiva-derived MSCs and verifying viability using trypan blue, 1.25x10^5^ MSCs were seeded per well in 6-well plates using 3 ml DMEM, supplemented as described above. After 24 hours of incubation, cell cycle synchronization (G1/G0 arrest) was performed by serum starvation by changing the medium to DMEM supplemented with 100μg/ml streptomycin and 100U/ml penicillin but lacking FBS.
Cell-cycle-synchronized MSCs from each sample were washed with 3% BSA buffer (supplemented with 0.09% sodium azide in 1xPBS) for hashing cells with oligonucleotide-tagged antibodies. Each of the 10 samples was labelled with 0.05 μg of a unique TotalSeq^TM^ A (0251-0260) anti-human Hashtag antibody (BioLegend, San Diego, CA, USA) for 30 minutes at 4° Celsius (Appendix Table 1). After washing the cells twice with 3% BSA buffer, MSCs were resuspended in 500μl 3% BSA buffer. Labeling MSCs samples with anti-CD298 and anti-β2-microglobulin Hashtag antibodies enabled the pooling of all 10 samples together for processing as a single sample because the antibody-linked oligonucleotides allow sample-specific demultiplexing. Single-cell preparation was conducted following the 10x Genomics Single Cell Protocols – Cell Preparation Guide. In brief, hash-tagged MSCs were thoroughly mixed and filtered using a 70μm cell strainer. After washing the MSCs twice with 1x Dulbecco's phosphate-buffered saline (Thermo Fisher Scientific) Solution (1xDPBS, without (w/o) calcium and magnesium and with 0.04% BSA), the total (living + dead) cell number was counted using cell counting chambers, and the proportion of dead cells was determined using trypan blue (Thermo Fisher Scientific). An appropriate volume of 1xDPBS was added per sample to reach the target cell concentration of 5000 MSCs in 5μl for each sample. 5μl of each of the 10 samples were pooled 1:1 to achieve 50.000 cells in 50μl 1xDPBS (w/o calcium and magnesium and with 0.04% BSA). The pooled sample was placed on ice and immediately transferred for single-cell library construction.

**Single-cell library construction and sequencing**

The Biomedical Sequencing Facility performed single-cell library construction and sequencing at the CeMM Research Center for Molecular Medicine of the Austrian Academy of Science. scRNA-seq libraries were constructed by the Chromium Controller using the Next GEM Single Cell 3' GEM, Library & Gel Bead Kit (v3.1, 10x Genomics, Pleasanton, CA, USA) according to the manufacturer’s instructions for a maximum cell recovery of 10,000 cells. Single-cell library sequencing was conducted using the Illumina NovaSeq 6000 platform.

**Demultiplexing, preprocessing, and analysis of scRNA-seq data**

The Core Facility Genomics of the Medical University of Vienna performed demultiplexing and pre-processing of raw sequencing data. The demultiplexing and further processing of the raw sequencing data were executed using the cellranger multi pipeline and count pipeline (v7.0.1., 10x Genomics) following the guidelines provided by 10x Genomics. FASTQ files were generated by running the cellranger mkfastq command within the cellranger multi pipeline. Hashtag oligonucleotides were used to assign the sample identity (tissue type and donor) to each cell barcode. To improve quality, cell barcodes not associated with any hashtag oligonucleotide or those related to several were specified as “no tag” or “multi-tag” (e.g. doublets), respectively, and excluded from downstream analysis. Additionally, non-cell associated reads were excluded by the cell calling algorithm within the cellranger multi and count pipeline. The sequenced reads were aligned to the GRCh38-2020-A assembly version, which functions as a human genome reference. Gene expression quantification in each cell was performed using the cellranger count pipeline, including intronic reads into the count matrix. A Loupe Browser output file was generated for subsequent analysis. A detailed summary of the sequencing output (sequencing details per donor and library information) is listed in Appendix Tables 3-6.

The Loupe Browser (v6.3.0., 10x Genomics) was used to illustrate demultiplexed data among all 10 samples by t-distributed stochastic neighbour embedding (t-SNE) projections. Re-clustering of PDL- and gingiva-derived MSCs per donor was based on the cell’s gene expression profiles and was depicted by t-SNE projections to investigate inter-tissue variability. Cell barcodes specific to “no tag” and “multi-tag” (e.g. doublets) were excluded from the clustering process. For quality control, the threshold by UMI counts per cell barcode, gene counts per cell barcode, and mitochondrial UMIs (% mitochondrial UMIs per barcode) were individually set for each clustering process per donor within the Loupe Browser. The mitochondrial UMI thresholds were based on the mitochondrial genes included in the selected reference genome (GRCh38/hg19). The 2D embedding-perplexity value for t-SNE was set to 30. Following Wang et al. (Wang et al. 2021) all cell clusters with more than 90% PDL- or gingiva-derived MSCs were identified as PDL- or gingiva-specific subpopulations, respectively. Cell clusters containing MSCs from the PDL and gingiva were termed “mixed.” The online tool “SCOPIT” (v1.1.4) was used to verify the required number of MSCs for single-cell RNA sequencing experiments (Davis et al. 2019). According to this tool, 229-507 cells per donor (depending on the donor) were required to detect at least 50 cells in each existing subpopulation with a 0.95 probability.

Differential gene expression (DGE) analysis was performed using the Seurat (V4) package (Satija et al. 2015; Butler et al. 2018; Stuart et al. 2019; Hao et al. 2021) in RStudio (v4.2.3.). First, cluster-specific cell barcodes and cluster annotations were extracted from the loupe file to filter the cellranger count pipeline output data in a cluster-specific manner, excluding clusters mainly consisting of mitotic cells. Each cell's measured gene expression data were normalized by the total expression using the global-scaling normalization method “LogNormalize” with a scale of 10,000. The 2000 most highly variable features were identified per dataset using the “FindVariableFeatures” command, and the gene expression data were linearly transformed using the “ScaleData” function. The “RunPCA” command was applied to run a linear dimensional reduction by principal component analysis (PCA) using the determined variable features as input. For cell clustering, the commands “FindNeighbors” and “FindClusters” were applied using the calculated dataset dimensionality (1-10 PCs) and the resolution parameter set to 0.8, respectively. The identity classes were set to user-defined cluster annotations to find the manually specified clusters by running the “Idents” function. Differentially expressed genes (DEGs) between tissue-specific subpopulations per donor (inter-tissue variability), and between PDL- or gingiva-specific subpopulations among all donors (donor-to-donor variability) were determined by the “FindAllMarkers” command using the Wilcoxon Rank Sum test. Only positive markers with an average log2FC of at least 0.25 and a minimum of 25% positive cells in the appropriate subpopulation were retained. Within the previously defined 2000 highly variable features per dataset, the top 10 or 20 differently expressed markers per subpopulation were selected. Expression heat maps for individual cells were created by running the “DoHeatmap” function. Features not included in the scaled datasets were omitted from the heat maps. In addition, the distribution of the expression probability of MSCs (ITGB1, MCAM, NT5E, THY1, and ENG) and hematopoietic (PTPRC, PECAM1, C34) surface markers across subpopulations was demonstrated by violin plots using the “VlnPlot” command.

**Pathway and process enrichment analysis**

To investigate functional differences between PDL- and gingiva-specific or between tissue-specific subpopulations of different donors, pathway and process enrichment analysis was conducted on highly differently (upregulated) expressed genes (p_val_adj < 0.05) using Metascape (Zhou et al. 2019). A cross-comparison of multiple input gene lists (unranked) was performed at the gene identity and ontology levels. Gene identity and functional overlaps are illustrated as circos plots. The GO Biological Processes, Reactome Gene Sets, and Canonical Pathways were used as ontology sources for pathway and process enrichment analysis. All genes in the human genome were used as enrichment background. Enriched terms with a minimum gene count of 3, an enrichment factor > 1.5, and a p-value < 0.01 were clustered predicated on their membership similarities. The cumulative hypergeometric distribution was used to calculate the p-values, whereas multiple hypothesis testing was corrected by the Benjamini-Hochberg Method (q-values). The kappa scores were calculated as a similarity metric for the hierarchical clustering of the enriched terms. A cluster comprises subtrees with a similarity > 0.3, and the enriched term with the highest p-value within a cluster was selected to typify the cluster. Because of multiple input gene lists, enrichment analysis was performed on each single input gene list, followed by combining the input gene lists into one list and applying enrichment analysis on this combined list. The best p-value among the individual input and combined gene lists was selected for reporting. Because this study was interested in pathways and processes enriched in selective cell subpopulations, enriched terms with diverse selective distributions were prioritized. The top 20 enriched clusters are presented as heatmaps showing their enrichment patterns among multiple cell clusters. Functional overlaps between multiple cell subpopulations are additionally demonstrated as circos plots using only significantly enriched ontology terms that include a maximum of 100 genes.
In addition, network plots were generated with Metascape (Zhou et al. 2019), in which enriched terms with a similarity > 0.3. are linked by edges. Within the top 20 enriched clusters, the enriched terms with the highest significance per enriched cluster were included in the enrichment networks. The creation of the network plots was restricted by the following settings: a maximum of 15 enriched terms per cluster and 250 enriched terms in total. The enrichment networks were visualized with a “force-directed” layout and annotated using Cytoscape (Shannon et al. 2003), where each node represents an enriched term. The single nodes depict pie charts whose size is proportional to the overall number of genes in the combined input gene lists that hit the specific enriched term. In contrast, the pie slices represent the percentage of genes in each input gene list that belong to a particular enriched term.

**Protein-protein network enrichment analysis**

Metascape (Zhou et al. 2019) was used to individually apply protein-protein interaction enrichment analysis to highly differently (upregulated) expressed genes for each subpopulation of interest. BioGrid, InWeb_IM, OmniPath, and STRING functioned as reference databases using physical interactions in BioGrid and STRING with a physical score < 0.132. To verify tightly linked network elements, the Molecular Complex Detection (MCODE) algorithm was separately executed on each protein-protein network that contained at least three and a maximum of 500 proteins. Each MCODE network was further investigated by pathway and process enrichment analysis, and the top three enriched terms (ranked by p-value) were used to describe the appropriate MCODE networks functionally.

**Extended results version**

**Exploring tissue-to-tissue heterogeneity**

*Gene signatures of tissue-specific subpopulations*

In the next step, we explored the gene signatures of the identified subpopulations in each donor separately to exclude donor-to-donor variability. The pairwise differential gene expression analysis after excluding mixed MSCs subsets revealed specific gene signatures for PDL- and gingiva-specific MSCs subpopulations in donor 2 (Figure 2A), 3 (Figure 2B), and 5 (Figure 2C). This analysis was not performed for donors 1 and 4 because they did not exhibit tissue-specific subpopulations in both gingiva and PDL.

The top 20 upregulated genes per tissue-specific subpopulations are depicted in heatmaps per donor (Figure 2D-I). In the PDL-specific subpopulation of donor 2, several significantly upregulated genes are related to cell growth (*IGFBP6, HTRA1, GPC6,* and *SFRP1*), cell motility/migration (*IGFBP6, FRMD5,* and *BCAR3*), development (*LAMA4*), differentiation (*PPARG,* and *LRMDA*), immunological (*SPON2, SLIT2, PI2F2, MAP4K4,* and *POU2F2*), and neurological (*CACNA1A, SLIT2*, and *CADPS*) processes, homeostatic control of intracellular metal (*MT2A*), the interaction between the cytoskeleton and (ECM) extracellular matrix (*SGCZ*), and cell morphology and cytoskeletal organization (*FMNL2*) (Figure 2D). The gingiva-specific subpopulation in donor 2 also showed significantly expressed genes involved in neurological processes (*CNTNAP2, ATP1B3, KIRREL3, NEGR1,* and *USP53)*. Additionally, significantly upregulated genes are involved in transcriptional processes (*HES1, NR4A3, LRRFIP1, RANBP2,* and *NFATC2*), ECM/tissue remodeling (*TIMP3,* and *MMP3*), regulating bone resorption (*TNFRSF11B*), calcium signaling (*AHNAK2)*, growth and apoptosis regulation (*GADD45B*), cellular stress response (*ATF3*), intracellular vesicle trafficking (*SNX9*), and adherence junctions (*RASSF8)* (Figure 2G).

In donor 3, several genes of the top 20 upregulated genes of the PDL-specific subpopulation are involved in neurological processes (*SLIT2, DOK6, NEGR1*, and *PENK)*, organogenesis, development, tissue differentiation, and body patterning (*GREM1, GREM2, KIF26B, TSHZ2, and NPAS3*), the organization of the actin cytoskeleton and cell polarity (*FMN2, and FREMD4A*). Single upregulated genes are related to the ECM (*ADAMTSL1*), bone formation, inflammation, wound healing (*PAPPA*), calcium ion import *(NALF1*), protein folding (*CLU*), vessel wall integrity (*COL8A1*), catabolic processes (*DPYD*), and several signaling pathways (*PRKG1*, and *EGFR*) (Figure 2E). Significantly upregulated genes of the gingiva-specific subpopulation can be assigned to cell differentiation, cell growth, apoptosis, angiogenesis (*S100A4, ADIRF, GPC6, ID1,* and *SFRP2*), neurological processes (*SNCA,* and *TNC)*, cytoskeleton, and cell adhesion (*ITGA8,* and *TUBA1A, TUBA1B, TUBB4B,* and *STMN1*), inflammation (*FABP5*), antimicrobial activity (*HMGN2*), zinc absorption (*CRIP1)*, heterochromatin formation (*H2AFZ*), transcription (*EPAS1*), and nucleotide metabolism (*NT5E*) (Figure 2H).

In donor 5, the top 20 upregulated genes of the PDL-specific subpopulation are related to processes in smooth muscles (*CALD1, TPM1,* and *TAGLN)*, neurological (*IL1RAPL1,* and *ARHGEF28)*, immunological (*LMO7*, and *ARHGEF28),* and developmental processes (*ZFPM2,* and *NRG1)*, to cell adhesion and migration (*NEXN, TGFBI, GAS6, TRIO, CDH6*, and *CCN2*). Additionally, upregulated genes are involved in the regulation of the transforming growth factor beta receptor signaling pathway (*SMURF2)*, chondrocyte proliferation and differentiation (*CCN2),* cross-linking of collagen and elastin (*LOX*), nitric oxide generation (*DDAH1)*, connective tissue microfibrils (*FBN2*), retina signal transduction *(PLCB4*), and calcium ion import (*NALF1*) (Figure 2F). Significantly upregulated genes of the gingiva-specific subpopulation can be assigned to fat cells (*ADIRF*) and other developmental processes (*PLEKHA5, TSH2, FRAS1, FMNL2*, and *ZNF2331)*, to cell differentiation, cell growth, apoptosis, angiogenesis (*S100A4*), cytoskeleton and ECM-associated processes *(PRKCE, SGCZ, and RGS2),* immunological processes (*SPON2)*, bone remodeling (*CTSK*), chondrocyte development, differentiation, proliferation (*TRPS1*), lipid metabolism (*PITPNC1*), zinc transport (*CRIP1*), cell polarity (*ARHGAP24*), and other transcriptional processes (*NFATC2*) (Figure 2I).

This indicates that PDL- and gingiva-specific MSCs’ subpopulations have differently expressed gene signatures. However, these results reveal functional overlaps between the two tissue-specific subpopulations.

*Exploring differences between tissue-specific and mixed MSCs’ subpopulations*

Next, we explored the differences between tissue-specific and mixed MSCs subpopulations per donor. After excluding proliferation-associated subpopulations (D1_mixed 5, D2_mixed 3, and D5_mixed 3), differential gene expression analysis revealed specific gene signatures for mixed and tissue-specific subpopulations, with minimal functional overlaps in donors 2, 4, and 5 (Appendix Figure 14-18A-C). Pathway and process enrichment analysis of the significantly upregulated genes identified numerous ontology clusters, including terms enriched in tissue-specific and mixed subpopulations. Other clusters of gene ontology terms were solely enriched in tissue-specific or mixed subpopulations: five and seven clusters in donor 2 (Appendix Figure 15C), twelve and four clusters in donor 3 (Appendix Figure 16C), one and eleven clusters in donor 4 (Appendix Figure 17C), and three and five clusters in donor 5 (Appendix Figure 18C). In donor 1, no tissue-specific subpopulations were detected (Appendix Figure 14). The most prominent observation was that each donor contained one mixed subpopulation (D1_mixed 3, D2_mixed 2, D3_mixed 1, D4_mixed 3, and D5_mixed 2), which was exclusively associated with translational-related processes, oxidative phosphorylation, mitochondrial, and respiratory electron transport (Appendix Figure 14C-18C). Additionally, this was verified by PPI network analysis (Appendix Figure 19-23).

**Exploring donor-to-donor heterogeneity of tissue-specific MSCs’ subpopulations**

*Donor-specific gene signatures in tissue-specific subpopulations*

Differential gene expression analysis revealed donor-specific gene signatures for both tissue-specific subpopulations. The top 10 differently expressed genes of this analysis are depicted in heatmaps in a tissue-specific manner (Figure 4C-D). Within the PDL-derived subpopulations of donor 2, significantly upregulated genes are related to cytoskeleton-associated processes, ECM organization, cell-substrate adhesion (*API3BP* and *SGCZ)*, neurological processes (*CACNA2D3*, and *RGS7*), immunological processes (*SPON2, CAV1*, and *ALCAM)*, adipogenic differentiation (*ADIRF*), and cell differentiation, motility, apoptosis, and angiogenesis (*S100A4*). In donor 3, the significantly upregulated genes of the PDL-specific subpopulation are associated with neurological processes (*GRIK2, BMERB1,* and *NPAS3*), microfibril formation (*FBN2*), hyaluronic acid metabolism (*CEMIP*), developmental processes (*GREM1*), protein turnover (*CTSB*), and cell adhesion (*IGFBP7*). The upregulated genes in the PDL-specific subpopulation of donor 5 are involved in processes related to cell growth, division, proliferation, and differentiation (*SFRP, GPC6, and FABP5*), retinoid homeostasis (*FBP1*, and *FABP5*), neurological processes (*PENK,* and *FABP5*), smooth muscle differentiation (*TAGLN*), cell adhesion (*POSTN*), and immunological processes (*IL1RAPL1,* and *FABP5*) (Figure 4C).

Within the gingiva-derived subpopulation of donor 2, significantly upregulated genes are related to neurological (*TENM2* and *SERPINE2*), ECM-associated (*MMP3* and *CCBE1*), and immunological processes (*CAV1* and *SYTL2)*, retina signal transduction (*PLCB4*), cytoskeleton organization (*RALGPS2*), fat metabolism and lymphopoiesis (*LEPR*) and bone resorption regulation (*TNFRSF11B*). In donor 3, upregulated genes of the gingiva-derived subpopulation are associated with neurological processes and development (*NLGN1* and *NES)*, cell growth, differentiation, migration, and angiogenesis (*PTN* and *SFRP2*), cell adhesion, cytoskeletal rearrangement (*ITGA8)*, vascular remodeling (*CTHRC1)*, and the regulation of cell cycle progression (*RGCC)*. Upregulated genes in the gingiva-specific subpopulation of donor 4 are involved in neurological processes (*CACNA2D3* and *CNTNAP2)*, cell proliferation, differentiation, growth, tissue repair, and embryonic development (*EGFR, GPC5, GPC6,* and *FGF7*), protection from oxidative stress (*MGST1*), and fatty acid biosynthesis (*SCD*). In donor 5, the upregulated genes of the gingiva-derived subpopulation are related to the following processes: neurological (*PENK* and *TCN)*, immunological (*SPON2*), and developmental (*FRAS1*) processes, adipogenic differentiation (*ADIRF*), the interaction between the ECM and cytoskeleton (*SGCZ*), and transcriptional regulation (*HMGA1*) (Figure 4D).

Together, these results suggest that the gene signatures of tissue-specific subpopulations differ depending on the donor.

**References**

Butler A, Hoffman P, Smibert P, Papalexi E, Satija R. 2018. Integrating single-cell transcriptomic data across different conditions, technologies, and species. Nat Biotechnol. 36(5):411–420. [accessed 2023 Jun 19]. <https://pubmed.ncbi.nlm.nih.gov/29608179/>.

Davis A, Gao R, Navin NE. 2019. SCOPIT: sample size calculations for single-cell sequencing experiments. BMC Bioinformatics. 20(1):1–6. [accessed 2024 Jun 11]. <https://bmcbioinformatics.biomedcentral.com/articles/10.1186/s12859-019-3167-9>.

Hao Y, Hao S, Andersen-Nissen E, Mauck WM, Zheng S, Butler A, Lee MJ, Wilk AJ, Darby C, Zager M, et al. 2021. Integrated analysis of multimodal single-cell data. Cell. 184(13):3573-3587.e29. [accessed 2023 Jun 19]. <https://pubmed.ncbi.nlm.nih.gov/34062119/>.

Satija R, Farrell JA, Gennert D, Schier AF, Regev A. 2015. Spatial reconstruction of single-cell gene expression data. Nat Biotechnol. 33(5):495–502. [accessed 2023 Jun 19]. <https://pubmed.ncbi.nlm.nih.gov/25867923/>.

Shannon P, Markiel A, Ozier O, Baliga NS, Wang JT, Ramage D, Amin N, Schwikowski B, Ideker T. 2003. Cytoscape: a software environment for integrated models of biomolecular interaction networks. Genome Res. 13(11):2498–2504. [accessed 2023 Jun 14]. https://pubmed.ncbi.nlm.nih.gov/14597658/.

Stuart T, Butler A, Hoffman P, Hafemeister C, Papalexi E, Mauck WM, Hao Y, Stoeckius M, Smibert P, Satija R. 2019. Comprehensive Integration of Single-Cell Data. Cell. 177(7):1888-1902.e21. [accessed 2023 Jun 19]. <https://pubmed.ncbi.nlm.nih.gov/31178118/>.

Viswanathan S, SHI Y, Galipeau J, Krampera M, Leblanc K, Martin I, Nolta J, Phinney DG, Sensebe L. 2019. Mesenchymal stem versus stromal cells: International Society for Cellular Therapy Mesenchymal Stromal Cell committee position statement on nomenclature. Cytotherapy. 21(10):1019–1024.

Wang Z, Chai C, Wang R, Feng Y, Huang L, Zhang Yiming, Xiao X, Yang S, Zhang Yunfang, Zhang X. 2021. Single-cell transcriptome atlas of human mesenchymal stem cells exploring cellular heterogeneity. Clin Transl Med. 11(12). [accessed 2023 Jun 9]. <https://pubmed.ncbi.nlm.nih.gov/34965030/>.

Zhou Y, Zhou B, Pache L, Chang M, Khodabakhshi AH, Tanaseichuk O, Benner C, Chanda SK. 2019. Metascape provides a biologist-oriented resource for the analysis of systems-level datasets. Nat Commun. 10(1). [accessed 2023 Jun 14]. https://pubmed.ncbi.nlm.nih.gov/30944313/.
